# Supplementary material for: Deep learning of left atrial structure and function provides link to atrial fibrillation risk
Source: Nat Commun. 2024 May 21;15:4304. doi: 10.1038/s41467-024-48229-w (PMC11109224; doi:10.1038/s41467-024-48229-w)
Supplement: Supplementary file 1 — Supplementary Information [file 41467_2024_48229_MOESM1_ESM.pdf]

Supplementary Note

Supplementary Note **1**

Supplementary Methods 3

Comparison with left atrial biplane measurements 3

GWAS sensitivity analysis: LVEDV-indexing 3

GWAS sensitivity analysis: no exclusion for abnormal cardiac filling patterns 4

GWAS sensitivity analysis: genetic diversity 4

Polygenic score sensitivity analyses 5

Supplementary Results 6

Semantic segmentation model quality assessment 6

Segmentation and reconstruction quality control 7

Comparison with left atrial biplane measurements 7

Quality control for the deep learning model for abnormal cardiac filling patterns 8

Relationship between cardiac filling patterns and left atrial volume 9

Atrial size was associated with AF, stroke, hypertension, and heart failure 9

GWAS sensitivity analysis - LVEDV-indexing 11

GWAS sensitivity analysis: no filtering for abnormal cardiac filling patterns 12

GWAS sensitivity analysis: genetic diversity 12

Supplementary Tables 14

Supplementary Table 1: relationship between left atrial measurements and prevalent disease 14

Supplementary Table 2: relationship between left atrial measurements and incident disease 15

Supplementary Table 3: relationship between left atrial measurements and incident disease after adjustment for left ventricular features 16

Supplementary Table 4: REML heritability and genetic correlation 17

Supplementary Table 5: ldsc heritability and intercept 18

Supplementary Table 6: genetic correlation between left atrial measurements, atrial fibrillation, and stroke 19

Supplementary Table 7: Relationship between atrial fibrillation polygenic score and left atrial measurements 20

Supplementary Table 8: relationship between left atrial polygenic scores and atrial fibrillation risk 21

Supplementary Figures **22**

Supplementary Figure 1 - Measurement distributions 22

Supplementary Figure 2 - Normal and abnormal cardiac filling patterns 23

Supplementary Figure 3 - Sample flow diagram 25

Supplementary Figure 4 - LV-adjusted left atrial phenotype Manhattan plots 26

Supplementary Figure 5 - Mendelian randomization method comparison plot for LAmin

vs atrial fibrillation 27
Supplementary Figure 6 - Pleiotropic associations for variants used in Mendelian randomization 29
Supplementary Figure 7 - Mendelian randomization method comparison plot for LAmin vs atrial fibrillation after removing 3 pleiotropic variants 30
Supplementary Figure 8 - Mendelian randomization method comparison plot for atrial fibrillation vs LAmin 32
Supplementary Figure 9 - Principal components of ancestry 34
Supplementary Figure 10 - Atrial volume with or without QC flags 35
Supplementary Figure 11 - Principal components of ancestry by inlier group 37
Supplementary References 38
FinnGen Consortium 40

Supplementary Methods

Comparison with left atrial biplane measurements

The UK Biobank's cardiovascular MRI imaging protocol did not include a volumetric short-axis stack throughout the left atrium[1], so left atrial measurements represent estimates of an unmeasured true left atrial volume. To assess quality, we compared the Poisson surface reconstruction approach with biplane measurements and tested each for association with prevalent atrial fibrillation. Using the R function *cor.test*, we correlated the Poisson surface reconstruction algorithm-based left atrial volume measurements with biplane-based volumes manually measured by experts[2].

GWAS sensitivity analysis: LVEDV-indexing

A sensitivity analysis was conducted to assess the consequence of accounting for body size based on each individual's LVEDV (rather than BSA). In addition to functioning as a sensitivity analysis for that purpose, accounting for left ventricular volume could, in principle, help to identify loci whose effects have the opposite effect direction between atrium and ventricle. However, adjusting for heritable covariates in GWAS can also induce associations via collider bias[3]. Like the primary analyses, the LVEDV-indexed sensitivity analyses were conducted with BOLT-LMM with the same covariates and settings (**Online Methods**). To attempt to identify LVEDV-indexed associations that were likely attributable to the adjustment for LVEDV, we also conducted a GWAS of LVEDV in the same participants with the same settings, and then tested each of the LVEDV-indexed lead SNPs for independent association with LVEDV.

GWAS sensitivity analysis: no exclusion for abnormal cardiac filling patterns

A sensitivity analysis was conducted to assess the consequence of retaining participants identified by the deep learning model as having apparently abnormal cardiac filling. For this sensitivity analysis, only LAmin and BSA-indexed LAmin were evaluated. Like the primary analyses, BOLT-LMM was used for this analysis with the same covariates and settings (**Online Methods**).

GWAS sensitivity analysis: genetic diversity

The primary analyses permitted the inclusion of all participants with LA measurements, regardless of genetic identity (**Supplementary Figure 9**). As a sensitivity analysis, individuals were analyzed within genetic inlier groups instead of jointly. To accomplish this, first self-reported ethnicity—which is only informally correlated with genetic identity—was aggregated into European (British, Irish, and Other European), African (African, “Any_other_Black_background”, “White_and_Black_African”, and “White_and_Black_Caribbean”), South Asian (Bangladeshi, Indian, Pakistani), and East Asian. Individuals with self-reported ancestry of “Any_other_mixed_background”, “Mixed”, “White_and_Asian”, “Any_other_Asian_background”, “Caribbean”, “Do_not_know”, “Other_ethnic_group”, or “Prefer_not_to_answer” were not analyzed further. Then, for each group of participants, the R package *aberrant* was run on the centrally computed genetic principal components of ancestry using a 40 standard deviation window similar to the approach of Bycroft, *et al*[4,5]. Inliers for each genetic identity group were retained. Individuals that were not part of an inlier genetic identity group were excluded. The genetic identity inlier groups were termed EUR, AFR, SAS, and EAS.

The sample sizes for the AFR, SAS, and EAS subsets were all well below the threshold recommended for the use of BOLT-LMM ("*We recommend BOLT-LMM for analyses of human genetic data sets containing more than 5,000 samples*", BOLT-LMM v2.4.1 User Manual https://alkesgroup.broadinstitute.org/BOLT-LMM/BOLT-LMM_manual.html). Therefore, for each of the four genetic inlier groups, a GWAS was conducted with REGENIE v2.2.4 which does not have the same limitation[6]. All models were adjusted for sex, age and age2 at the time of MRI, the first 10 principal components of ancestry, the genotyping array, and the MRI scanner's unique identifier. Fixed-effect meta-analysis was then conducted with METAL (release version 2020-05-05) [7].

Two additional GWAS were conducted in BOLT-LMM v2.3.4 using the same covariates as the primary GWAS: one for the inlier EUR population, and another where an equivalent number of individuals were dropped at random from the original GWAS cohort (without regard for genetic inlier grouping) to yield a sample size that was the same as the inlier EUR population.

GWAS loci from the primary analysis were fetched from the meta-analysis, the EUR-specific GWAS, and the GWAS in which individuals were dropped at random.

Polygenic score sensitivity analyses

In addition to the primary LA polygenic scores produced with PRScs, an additional set of LA polygenic scores was created as a weighted allelic sum based on the lead variants for each trait. That is, for each tested participant, at each of the lead variant alleles, the number of effect alleles possessed by the participant was multiplied by the effect estimate; these were then summed for all alleles for each phenotype. They were tested for association with diseases in the same way as the PRScs scores.

Supplementary Results

Semantic segmentation model quality assessment

In a held-out test set of 20 manually annotated images from the two-chamber short axis view that were not used in training or validation, the average Dice coefficient was 0.89 (SD 0.06) for the left atrial blood pool. For 20 held-out images from the three-chamber view, the Dice score was 0.88 (SD 0.07). For 40 held-out images from the four-chamber view, the Dice score was 0.94 (SD 0.03).

The short axis imaging sequence was not designed to capture the atria: the atrial short axis sequence was eliminated from the acquisition protocol to save acquisition time[1]. The left atrium was nevertheless recognizable in the basal-most segments of images obtained in the short axis view. In the short axis view, the average Dice score for the left atrium was 0.78 (SD 0.35) when weighted by the total number of pixels assigned to the left atrium by the cardiologist or the model, or 0.90 (SD 0.28) when considering images correctly identified by the model as having no left atrial pixels to have a Dice score of 1.

In the two-chamber view, the average Hausdorff distance was 6.7mm (SD 4.0mm). In the three-chamber view, the average Hausdorff distance was 8.8mm (SD 8.5mm). In the four-chamber view, the average Hausdorff distance was 5.2mm (SD 4.1mm). In the short-axis view, the average Hausdorff distance was 5.8mm (SD 4.2mm).

In the two chamber view, the average mean contour distance was 1.8mm (SD 0.6mm). In the three-chamber view, the average mean contour distance was 2.3mm (SD 2.2mm). In the four-chamber view, the average mean contour distance was 1.3mm (SD 0.90mm). In the short-axis

view, the average mean contour distance was 1.7mm (SD 1.7mm). The mean contour distance for the automated left atrial segmentation in each of these views was less than the in-plane pixel spacing of 1.83mm.

Segmentation and reconstruction quality control

QC-flagged samples (due to more than 1 connected component, frame-to-frame pixel changes greater than 5 standard deviations above the mean, the absence of left atrial pixels, or an abnormal number of CINE images as detailed in the **Online Methods**) were significantly more likely to fail to achieve a successful Poisson reconstruction (OR 1.4, P=1.3E-19). Among the left atria that were successfully reconstructed, we tested whether the presence or absence of any of the QC flags was associated with volumetric measurements. However, the distribution was similar regardless of QC status (**Supplementary Figure 10**); the presence of QC flags was statistically non-significant for LAmin (0.020 SD greater with a flag, P=0.06) and had a similarly small effect estimate for LAmax (0.036 SD greater with a flag, P=5E-04). Therefore, all samples that were successfully reconstructed were retained for analysis.

Comparison with left atrial biplane measurements

We correlated the Poisson surface reconstruction algorithm-based left atrial volume measurements with biplane-based volumes manually measured by experts in 3,401 participants[2]. When limiting the inputs into the Poisson surface reconstruction algorithm to only the two- and four-chamber long axis views ("Poisson biplane"), which are the two views used to calculate the biplane volume, the correlation improved for both LAmax (from r=0.814, 95% CI 0.802 to 0.825, P=2.9E-804 with the full reconstruction to r=0.887, 95% CI 0.880 to 0.894, P=4.5E-1143 with the Poisson biplane) and LAmin (from r=0.768, 95% CI 0.754 to 0.781, P=1.1E-659 to r=0.860, 95% CI 0.851 to 0.868, P=6.9E-994). We interpreted these results as

supporting the notion that, when presented with the same input information, the modeling approach yields estimates that are similar to the standard biplane estimation.

We then used logistic regression to recapitulate prior observations that individuals with pre-existing atrial fibrillation have larger atrial volumes[8,9]. In a subset of 39,148 participants, of whom 808 had atrial fibrillation, both the full Poisson reconstruction and the Poisson biplane reconstruction could be performed. Although the Poisson biplane better correlated with the manual measurements in the previous analysis, the full Poisson reconstruction was more strongly associated with prevalent atrial fibrillation (LAmax OR 1.72, P=1.3E-78 and LAmin OR 1.86, P=1.0E-132) compared to the Poisson biplane model (LAmax OR 1.65, P=6.3E-66 and LAmin OR 1.80, P=2.8E-130).

We interpreted these findings as indicating that (1) the Poisson-based measurements were well correlated with manual measurements, and (2) while full volumetric imaging stacks through the atria were not available to adjudicate correctness, the Poisson-based measurements that incorporated all available views (2ch, 3ch, 4ch, and SAX) were more strongly correlated with atrial fibrillation than the Poisson biplane measurements.

Quality control for the deep learning model for abnormal cardiac filling patterns

Among 200 participants whose MRIs were manually reviewed (100 flagged as having abnormal cardiac filling patterns and 100 flagged as having normal cardiac filling patterns), manual review determined that 164 were normal and 36 were abnormal. The sensitivity of the model for identifying abnormal cardiac filling patterns was 100% (95% CI 90.3-100.0%) and the specificity was 61% (95% CI 53.1-68.5%). These findings suggested that the model may have over-

detected abnormal cardiac filling—leading to the exclusion of more participants than necessary—but had little evidence for false negatives.

Relationship between cardiac filling patterns and left atrial volume

Among the 40,558 participants with LA measurements whose filling patterns could be analyzed, we identified 1,013 participants whose patterns did not appear to be consistent with normal cardiac filling patterns. Of these, 376 (37%) had a pre-existing history of AF or atrial flutter. The same 376 participants represented 32% of all 1,189 participants with a history of AF or atrial flutter. The remaining 637 participants with abnormal cardiac filling patterns did not have a history of AF or flutter, representing only 1.6% of the 39,369 participants without such history.

Among participants with no history of AF or atrial flutter, those with an abnormal atrial cardiac filling patterns had significantly elevated LA volumes (**Figure 3**; N = 637; LAmin: +1.3 standard deviations [SD] compared to the 38,732 with no AF history and normal cardiac filling patterns, P = 3.1E-321; LAmax: +0.8 SD, P = 3.7E-103). The most extreme volumes were observed in participants with a history of AF or atrial flutter who also had an abnormal cardiac filling pattern (N = 376; LAmin: +4.3 SD compared to those with no AF history and normal cardiac filling patterns, P = 1.6E-1937; LAmax: +2.5 SD, P = 8.9E-623). The 813 participants with a history of AF and normal cardiac filling patterns had larger volumes than those with normal cardiac filling patterns and no AF history (LAmin: +0.6 SD, P = 2.4E-101).

Atrial size was associated with AF, stroke, hypertension, and heart failure

After excluding participants with abnormal cardiac filling patterns, we conducted analyses in the remaining 39,545 participants. First, we confirmed previous reports of the relationship between prevalent diseases and atrial size and function. Compared to the 38,732 UK Biobank participants without a diagnosis of AF or atrial flutter prior to MRI, the 813 with a pre-existing

diagnosis had larger LA volumes (LAmin: +8.8mL, P = 9.2E-117; LAmax: +10.1mL, P = 1.5E-61) and a reduced LAEF (-4.6%, P = 9.7E-68). Participants with a history of heart failure, hypertension, or stroke also had elevated LA volumes (**Figure 3, left panel**; **Supplementary Table 1**).

We then examined the relationship between LA measurements and incident cardiovascular diseases. We excluded an additional 1,114 participants with prevalent AF, heart failure, or stroke diagnosed prior to MRI, and 1,525 with missing height, weight, or body mass index (BMI) measurements at the time of MRI. Only a brief period of follow-up time of 2.2 +/- 1.5 years after the MRI assessment center visit was available for most participants. Nevertheless, participants with a larger LA had a greater risk of subsequently being diagnosed with AF (293 incident AF diagnoses; hazard ratio [HR] 1.73 per standard deviation [SD] increase in LAmin; 95% CI 1.60-1.88; P = 4.0E-39; **Figure 3, right panel**). The LAmin was also associated with an increased risk of incident ischemic stroke (98 cases; HR 1.32 per SD; 95% CI 1.11-1.57; P = 2.0E-03) and heart failure (125 cases; HR 1.69; 95% CI 1.48-1.92; P = 1.3E-15). The associations between other LA measurements and these diseases are detailed in **Supplementary Table 2**.

We performed a sensitivity analysis that accounted for ECG features and left ventricular structure and function; this yielded a similar point estimate for LAmin as a marker of incident AF risk (HR 1.89 per SD; 95% CI 1.66-2.15; P = 4.5E-22). In this sensitivity analysis, LAmin remained a significant predictor of incident heart failure (HR 1.51 per SD; 95% CI 1.23-1.86; P = 8.1E-05) but not of incident ischemic stroke (HR 1.10 per SD; 95% CI 0.84-1.43; P = 0.48; **Supplementary Table 3**).

GWAS sensitivity analysis - LVEDV-indexing

We are not aware of a general solution to the interpretation of GWAS signals that incorporate adjustment for heritable covariates. However, we observed the LVEDV-indexed lead SNPs to fall into three patterns: first, some SNP associations appeared to be driven largely by the LVEDV indexing rather than LA volume. As an example of this pattern, the LVEDV-indexed LAmax association with *BAG3* (P=3.5E-10) was comparable to that for the LVEDV association with *BAG3* alone (P=2.1E-10), while the unadjusted LAmax measurement was not associated (P > 1E-3). At each of these loci, the effect direction in LVEDV was opposite to that in the respective LVEDV-indexed LA volume GWAS, which was expected. Practically, these signals appeared to be driven by the LVEDV values, with the LA measurements acting as noise. Second, some SNP associations appeared to be driven by the LAmax association alone, with only minimal contribution from the LVEDV adjustment. For example, the LVEDV-indexed LAmax association with *IRAK1BP1* (P=2.0E-8) was similar to that for the LAmax association (P=2.7E-11), while the SNP was not associated with LVEDV (P > 1E-3). Third, some SNP associations appeared to be driven by the interplay between LA volumes and the LVEDV adjustment. For example, the *NEDD4L* locus was associated with LVEDV-indexed LAmax (P=4.7E-8) despite not being strongly associated with either LVEDV or LAmax alone (P > 1E-3 for both).

For the LVEDV-indexed LA volumes, 11 loci reached genome-wide significance for LAmax, 12 for LAmin, and four for LASV. Of these, six of the LVEDV-indexed LAmax loci had association P < 1E-3 with LVEDV, as did nine of the LAmin loci and two of the LASV loci. Novel loci that were not associated at genome-wide significance in the unadjusted GWAS, and which were not associated with LVEDV at 1E-3 or stronger, included *BLK*, *ANKRD1*, *MYH7*, and *NEDD4L* for LAmax; *CASQ2*, *DHX15*, *PROB1*, *UQCRB*, *ANKRD1*, and *MYH7* for LAmin, and *TNKS* and *HNRNPM* for LASV. Most of these loci were identified in the BSA-indexed GWAS as well.

GWAS sensitivity analysis: no filtering for abnormal cardiac filling patterns

Given the high sensitivity but low specificity of the model detecting abnormal cardiac filling patterns, sensitivity analysis retained the 615 participants who were not identified as having a normal cardiac filling pattern for GWAS of LAmin and BSA-indexed LAmin, yielding a total sample size of N=35,664 participants. (Because some participants are excluded by other criteria downstream of this filter in the primary GWAS, this number is smaller than the 1,013 noted in **Supplementary Figure 3**.) The lead SNPs are recorded in **Supplementary Data 11**. Compared with the main analysis of 35,049 participants, some loci with marginal P-values were lost while others were gained; net, an additional two loci (10 in total) were identified for LAmin and an unchanged number of loci (13) were significant for BSA-indexed LAmin. For example, the association signal for *PITX2* variant rs2466455 for LAmin increased in significance from P=4.6E-06 to P=3.10E-08 in this sensitivity analysis. Similarly the strongest associated variant near *PITX2* for BSA-indexed LAmin in this analysis (rs2723334, P=1.70E-10) had stronger evidence for association than in the primary analysis (P=2.2E-08).

GWAS sensitivity analysis: genetic diversity

Data from all participants were used for the primary GWAS, incorporating a diversity of genetic identities (**Supplementary Figure 9**). In a sensitivity analysis, only individuals with inlier genetic identities for one of four inlier groups were retained and analyzed separately (EUR, AFR, SAS, or EAS; **Supplementary Figure 11**). In this analysis, the largest inlier group was that for EUR, with 31,878 participants (9.9% smaller than the primary analysis). The second largest group was comprised of the 2,655 participants (7.6%) who were not genetic inliers for any group and were therefore not included in these sensitivity analyses. This was followed by SAS (N=284), AFR (N=133), and EAS (N=99), together comprising about 1.5% of the primary GWAS sample

size. GWAS were separately conducted for EUR, SAS, AFR, and EAS, and then meta-analyzed. Because of the loss of the participants who were included in the joint analysis but were not inliers for any genetic identity group, the multi-ancestry meta-analytic approach represented a loss of 7.6% of the total sample size compared to the primary analysis. These meta-analytic P-values were fetched for the lead variants from the primary analysis and are displayed in **Supplementary Data 2** as the "*P_META*" column.

Two additional sensitivity analyses were performed using BOLT-LMM: a EUR-specific GWAS, and an analysis in which individuals were dropped at random to achieve the same sample size as the EUR-specific GWAS. The P-values for the primary analysis's lead variants are also displayed in **Supplementary Data 2** with the "*P_EUR*" and "*P_RANDOMDROP*" columns, respectively.

The weakest association signal occurred for the BSA-indexed LAmin phenotype in the multi-ancestry meta-analysis at the *GOSR2* locus (P=2.5E-06), which was an order of magnitude weaker than the evidence for the EUR subgroup without meta-analysis (P=2.0E-07). Nevertheless, across these sensitivity analyses, we largely observed minor variation in association signal without clear evidence for population stratification.

Supplementary Tables

Supplementary Table 1: relationship between left atrial measurements and prevalent disease

Estimate	SE	T	P	Trait	Condition	N tested	N with disease
10.1	0.6	16.6	1.5E-61	LAmax	Prevalent AF	39545	813
1.9	1.4	1.3	1.9E-01	LAmax	Prevalent Stroke	39545	149
8.7	1.2	7.3	3.4E-13	LAmax	Prevalent CHF	39544	210
4.6	0.2	24.0	1.5E-126	LAmax	Prevalent HTN	39545	11852
8.8	0.4	23.0	9.2E-117	LAmin	Prevalent AF	39545	813
2.4	0.9	2.7	6.7E-03	LAmin	Prevalent Stroke	39545	149
7.7	0.7	10.3	7.3E-25	LAmin	Prevalent CHF	39544	210
2.5	0.1	20.7	2.4E-94	LAmin	Prevalent HTN	39545	11852
-4.6	0.3	-17.4	9.7E-68	LAEF	Prevalent AF	39545	813
-1.3	0.6	-2.2	2.9E-02	LAEF	Prevalent Stroke	39545	149
-3.8	0.5	-7.3	3.0E-13	LAEF	Prevalent CHF	39544	210
-0.4	0.1	-4.9	7.5E-07	LAEF	Prevalent HTN	39545	11852

Association between left atrial measurements (dependent variables) and condition present at the time of imaging (independent variables). Models were adjusted for age, sex and the magnetic resonance imaging device serial number. Effect estimates and standard errors are displayed in standard deviation units. P values are two-tailed. SE: standard error.

Supplementary Table 2: relationship between left atrial measurements and incident disease

Coef	HR	SE (Coef)	Z	P	Trait	Condition	N tested	N with disease	Mean survival	SD survival
0.48	1.61	0.05	9.00	2.2E-19	LAmax	Incident AF	36900	293	2.2	1.5
0.27	1.31	0.10	2.68	7.3E-03	LAmax	Incident Stroke	36900	98	2.3	1.5
0.45	1.57	0.08	5.49	3.9E-08	LAmax	Incident CHF	36887	125	2.3	1.5
0.14	1.15	0.05	2.83	4.7E-03	LAmax	Incident HTN	26088	469	2.2	1.5
0.55	1.73	0.04	13.09	4.0E-39	LAmin	Incident AF	36900	293	2.2	1.5
0.28	1.32	0.09	3.09	2.0E-03	LAmin	Incident Stroke	36900	98	2.3	1.5
0.52	1.69	0.07	8.00	1.3E-15	LAmin	Incident CHF	36887	125	2.3	1.5
0.20	1.22	0.05	4.31	1.6E-05	LAmin	Incident HTN	26088	469	2.2	1.5
-0.63	0.53	0.06	-10.86	1.9E-27	LAEF	Incident AF	36900	293	2.2	1.5
-0.23	0.80	0.10	-2.22	2.7E-02	LAEF	Incident Stroke	36900	98	2.3	1.5
-0.57	0.56	0.09	-6.50	8.2E-11	LAEF	Incident CHF	36887	125	2.3	1.5
-0.18	0.84	0.05	-3.77	1.7E-04	LAEF	Incident HTN	26088	469	2.2	1.5

Association between left atrial measurements (independent variables) and incidence of conditions subsequent to imaging (dependent variables) based on Cox proportional hazards models. Models were adjusted for age, sex, the magnetic resonance imaging device serial number, height, weight, and body mass index. P values are two-tailed. Effect estimates (“Coef”) are exponentiated to hazard ratios (“HR”). SE: standard error.

Supplementary Table 3: relationship between left atrial measurements and incident disease after adjustment for left ventricular features

Coef	HR	SE (Coef)	Z	P	Trait	Condition	N tested	N with disease	Mean survival	SD survival
0.63	1.87	0.09	7.06	1.7E-12	LAmax	Incident AF	36900	293	2.2	1.5
-0.06	0.94	0.17	-0.34	7.4E-01	LAmax	Incident Stroke	36900	98	2.3	1.5
0.36	1.44	0.14	2.57	1.0E-02	LAmax	Incident CHF	36887	125	2.3	1.5
0.27	1.31	0.08	3.55	3.9E-04	LAmax	Incident HTN	26088	469	2.2	1.5
0.63	1.89	0.07	9.66	4.5E-22	LAmin	Incident AF	36900	293	2.2	1.5
0.10	1.10	0.14	0.70	4.8E-01	LAmin	Incident Stroke	36900	98	2.3	1.5
0.41	1.51	0.11	3.94	8.1E-05	LAmin	Incident CHF	36887	125	2.3	1.5
0.27	1.30	0.06	4.27	1.9E-05	LAmin	Incident HTN	26088	469	2.2	1.5
-0.61	0.54	0.08	-8.14	3.8E-16	LAEF	Incident AF	36900	293	2.2	1.5
-0.16	0.85	0.13	-1.31	1.9E-01	LAEF	Incident Stroke	36900	98	2.3	1.5
-0.42	0.66	0.11	-3.75	1.8E-04	LAEF	Incident CHF	36887	125	2.3	1.5
-0.17	0.84	0.06	-2.89	3.9E-03	LAEF	Incident HTN	26088	469	2.2	1.5

Association between left atrial measurements (independent variables) and incidence of conditions subsequent to imaging (dependent variables) based on Cox proportional hazards models. Models were adjusted for age, sex, the magnetic resonance imaging device serial number, height, weight, body mass index, heart rate, electrocardiographic features (P-wave duration, QRS duration, PQ interval, QTc interval) and left ventricular features (end-systolic volume, end-diastolic volume, and ejection fraction). P values are two-tailed. Effect estimates ("Coef") are exponentiated to hazard ratios ("HR"). SE: standard error.

Supplementary Table 4: REML heritability and genetic correlation

**Trait 1**	**Trait 2**	**Corr**	**SE**	**Meaning**
LAEF		0.14	0.02	heritability
LAmax		0.37	0.02	heritability
LAmax_indexed		0.32	0.02	heritability
LAmin		0.33	0.02	heritability
LAmin_indexed		0.27	0.02	heritability
LASV		0.28	0.02	heritability
LASV_indexed		0.22	0.02	heritability
LAEF	LAmax_indexed	-0.42	0.06	genetic correlation
LAEF	LAmin_indexed	-0.71	0.03	genetic correlation
LAEF	LASV	-0.14	0.07	genetic correlation
LAEF	LASV_indexed	0.00	0.07	genetic correlation
LAmax	LAEF	-0.48	0.05	genetic correlation
LAmax	LAmax_indexed	0.90	0.01	genetic correlation
LAmax	LAmin	0.95	0.01	genetic correlation
LAmax	LAmin_indexed	0.88	0.01	genetic correlation
LAmax	LASV	0.93	0.01	genetic correlation
LAmax	LASV_indexed	0.77	0.02	genetic correlation
LAmax_indexed	LAmin_indexed	0.94	0.01	genetic correlation
LAmax_indexed	LASV_indexed	0.91	0.01	genetic correlation
LAmin	LAEF	-0.72	0.03	genetic correlation
LAmin	LAmax_indexed	0.86	0.01	genetic correlation
LAmin	LAmin_indexed	0.94	0.01	genetic correlation
LAmin	LASV	0.77	0.03	genetic correlation
LAmin	LASV_indexed	0.62	0.04	genetic correlation
LAmin_indexed	LASV_indexed	0.72	0.04	genetic correlation
LASV	LAmax_indexed	0.84	0.02	genetic correlation
LASV	LAmin_indexed	0.70	0.04	genetic correlation
LASV	LASV_indexed	0.87	0.01	genetic correlation

REML-based heritability of each trait and genetic correlation between trait pairs are depicted. Corr represents the point estimate for each estimate. SE: standard error.

Supplementary Table 5: *ldsc* heritability and intercept

Name	Observed scale h2	Lambda GC	Mean χ^2	Intercept	Ratio
invnorm_LAEF_poisson	0.1078 (0.0224)	1.0496	1.0649	0.9983 (0.0093)	< 0
invnorm_LAmax_poisson	0.2351 (0.0274)	1.1301	1.1496	0.989 (0.0106)	< 0
invnorm_LAmin_poisson	0.2014 (0.0212)	1.1175	1.1315	0.9963 (0.0087)	< 0
invnorm_LASV_poisson	0.1891 (0.0313)	1.0926	1.1085	0.9816 (0.0126)	< 0
invnorm_LAmax_poisson_indexed	0.2361 (0.0298)	1.1459	1.1409	0.9914 (0.0112)	< 0
invnorm_LAmin_poisson_indexed	0.2157 (0.0227)	1.0957	1.1317	0.9979 (0.009)	< 0
invnorm_LASV_poisson_indexed	0.193 (0.0339)	1.0957	1.0993	0.9812 (0.0133)	< 0

Ldsc-based heritability, lambda GC, mean χ^2, and *ldsc* intercepts are depicted.

Supplementary Table 6: genetic correlation between left atrial measurements, atrial fibrillation, and stroke

LA Measurement	Disease	Genetic correlation	SE	Z	P
LAEF	AF	-0.16	0.08	-2.0	5.1E-02
LAEF	All stroke	-0.14	0.10	-1.3	1.8E-01
LAEF	Cardioembolic stroke	-0.16	0.28	-0.6	5.8E-01
LAmax	AF	0.32	0.06	5.2	1.6E-07
LAmax	All stroke	0.17	0.07	2.4	1.8E-02
LAmax	Cardioembolic stroke	0.13	0.24	0.6	5.7E-01
LAmax_indexed	AF	0.26	0.06	4.5	6.7E-06
LAmax_indexed	All stroke	0.14	0.07	2.0	4.1E-02
LAmax_indexed	Cardioembolic stroke	0.11	0.19	0.6	5.8E-01
LAmin	AF	0.37	0.06	6.4	2.0E-10
LAmin	All stroke	0.21	0.08	2.5	1.2E-02
LAmin	Cardioembolic stroke	0.18	0.31	0.6	5.7E-01
LAmin_indexed	AF	0.33	0.06	5.8	7.7E-09
LAmin_indexed	All stroke	0.19	0.08	2.4	1.7E-02
LAmin_indexed	Cardioembolic stroke	0.15	0.27	0.6	5.8E-01
LASV	AF	0.18	0.06	3.1	2.0E-03
LASV	All stroke	0.10	0.07	1.4	1.7E-01
LASV	Cardioembolic stroke	0.07	0.14	0.5	6.0E-01
LASV_indexed	AF	0.09	0.06	1.5	1.3E-01
LASV_indexed	All stroke	0.04	0.06	0.7	4.8E-01
LASV_indexed	Cardioembolic stroke	0.02	0.06	0.4	6.7E-01

LA: left atrium. SE: standard error. P values are two-tailed.

Supplementary Table 7: Relationship between atrial fibrillation polygenic score and left atrial measurements

Estimate	Low	High	SE	T	P	Score	Trait	N
0.039	0.029	0.048	0.0049	7.93	2.3E-15	af.prs	LAmax	35049
0.042	0.032	0.053	0.0054	7.85	4.3E-15	af.prs	LAmax_indexed	33893
0.052	0.042	0.061	0.0049	10.49	1.1E-25	af.prs	LAmin	35049
0.055	0.045	0.065	0.0053	10.43	2.1E-25	af.prs	LAmin_indexed	33893
-0.047	-0.057	-0.037	0.0052	-9.03	1.8E-19	af.prs	LAEF	35049
0.013	0.003	0.023	0.0050	2.61	9.1E-03	af.prs	LASV	35049
0.012	0.001	0.023	0.0054	2.20	2.8E-02	af.prs	LASV_indexed	33893

Low and High represent the lower and upper bounds of the 95% confidence interval for the Estimate. SE: standard error. Units are in standard deviations of the left atrial measurements per standard deviation of the atrial fibrillation polygenic score. P values are two-tailed from a linear model.

Supplementary Table 8: relationship between left atrial polygenic scores and atrial fibrillation risk

N	Disease	Score	N with Disease	Beta	HR	SE	P
417881	Atrial fibrillation or flutter	invnorm_LAmax	21147	0.057	1.06	0.007	2.5E-16
417881	Atrial fibrillation or flutter	invnorm_LAmax_indexed	21147	0.066	1.07	0.007	1.4E-21
417881	Atrial fibrillation or flutter	invnorm_LAmin	21147	0.078	1.08	0.007	1.7E-29
417881	Atrial fibrillation or flutter	invnorm_LAmin_indexed	21147	0.082	1.09	0.007	7.4E-32
417881	Atrial fibrillation or flutter	invnorm_LASV	21147	0.026	1.03	0.007	2.6E-04
417881	Atrial fibrillation or flutter	invnorm_LAEF	21147	-0.059	0.94	0.007	8.3E-18

HR: hazard ratio in atrial fibrillation risk per standard deviation in the left atrial polygenic score. SE: standard error. P values are two-tailed from a Cox model.

Supplementary Figures

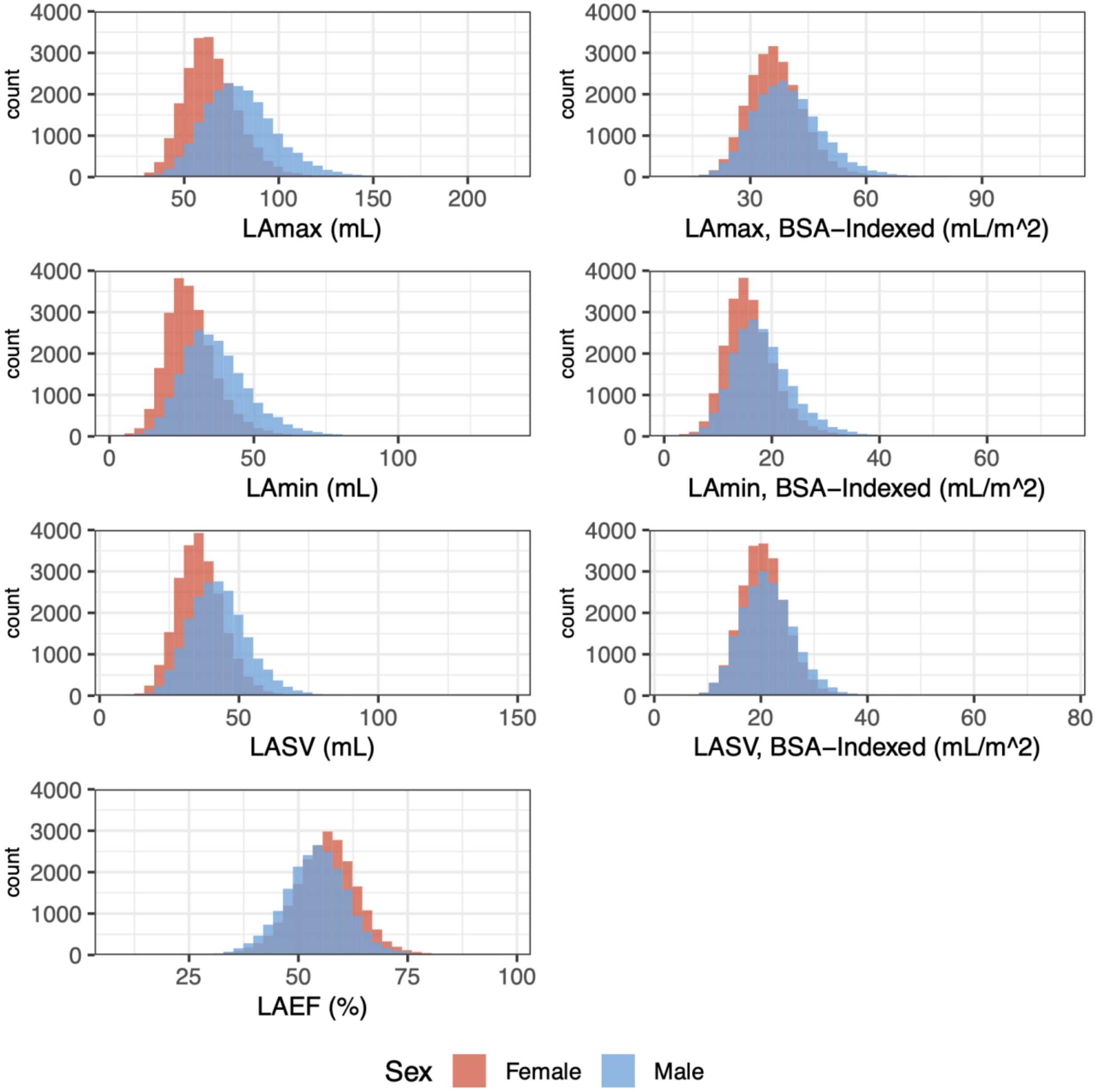

Supplementary Figure 1 - Measurement distributions

Trait distributions for the left atrial phenotypes without adjustment and after adjustment for body surface area (BSA). LAEF is dimensionless and is therefore not adjusted for BSA.

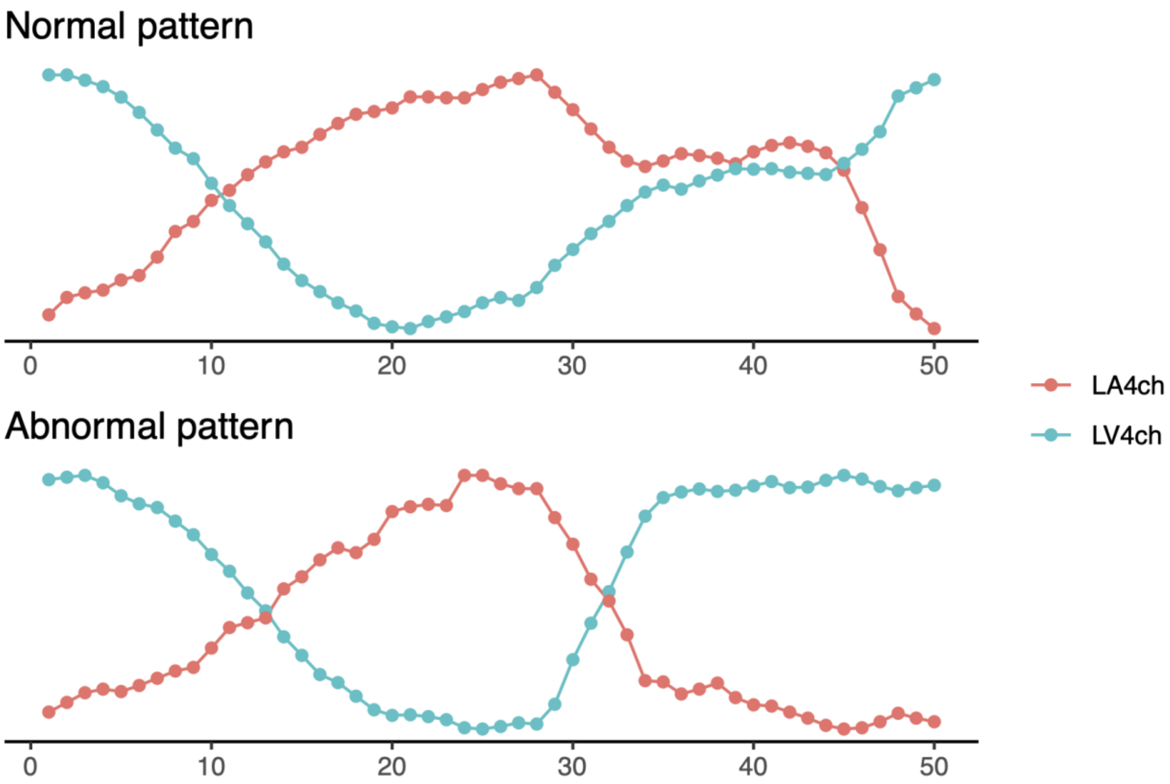

Supplementary Figure 2 - Normal and abnormal cardiac filling patterns

Curves depicting the data used in the abnormal filling pattern detector are displayed for one individual with a normal pattern (**top panel**) and one with an abnormal pattern (**bottom panel**). For visual simplicity, only the left atrial and left ventricular curves from the four-chamber view are displayed. Each datum represents the cross-sectional area at each time point for each chamber. Values are scaled between 0 and 1 (y-axis) on a per-chamber basis so that the maximum is always 1 and the minimum is always 0 for each chamber independently, which is consistent with how the data are transformed prior to being input into the deep learning model. Values are visualized at the 50 timepoints during image acquisition (x-axis). Both panels begin at ventricular end-diastole. The example in the **top panel** reveals a triphasic pattern: ventricular systole continues until timepoint 20, passive ventricular filling until timepoint 45, and then an active ventricular filling phase due to atrial systole from 45-50. The example in the **bottom**

panel reveals a biphasic pattern: there is only ventricular systole until timepoint ~25 and ventricular diastole for the remainder of the cycle, with the atrium passively filling and emptying in parallel.

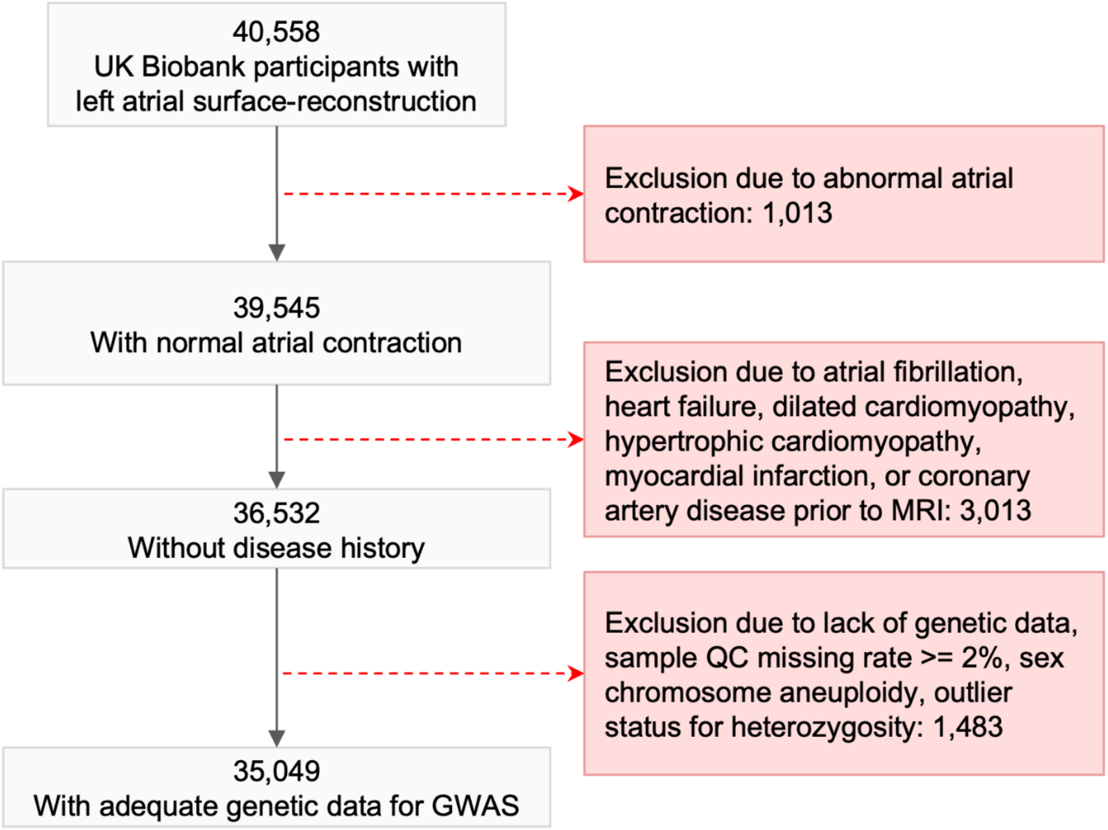

Supplementary Figure 3 - Sample flow diagram

Sample exclusion steps between surface reconstruction and the creation of the GWAS cohort are described.

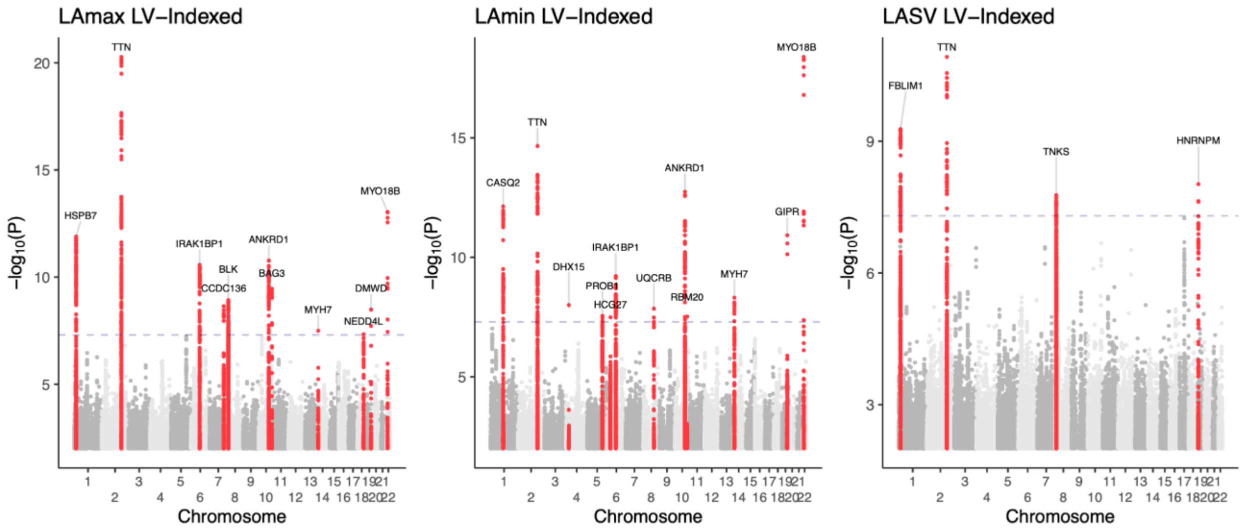

Supplementary Figure 4 - LV-adjusted left atrial phenotype Manhattan plots

Manhattan plots for LA measurements divided by LVEDV. X-axis: chromosomal position. Y-axis: -log10(P-value). Nearest gene names are annotated near significant loci, which are colored in red.

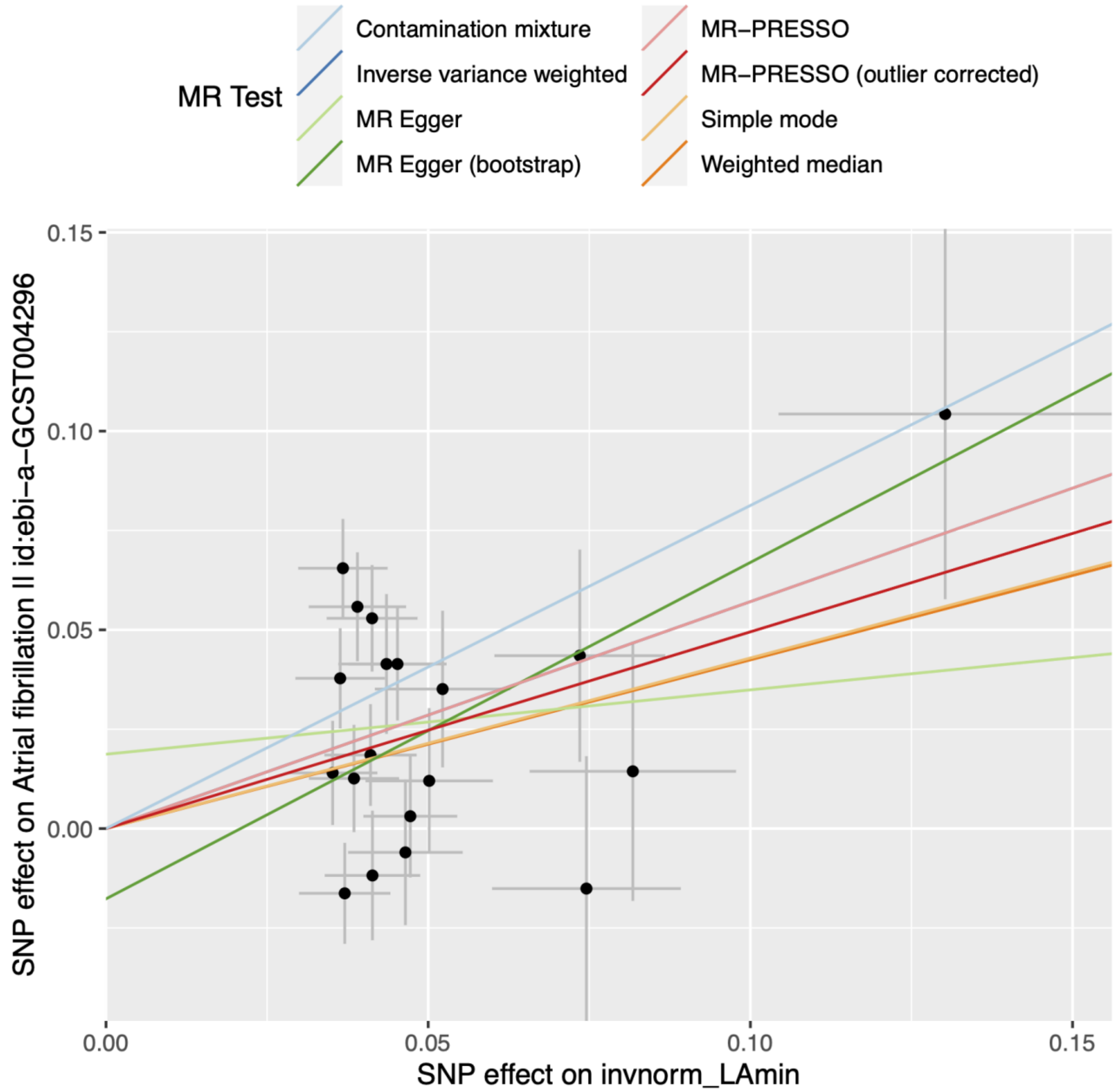

Supplementary Figure 5 - Mendelian randomization method comparison plot for LAmin vs atrial fibrillation

SNP effects on the exposure (X-axis) are plotted against SNP effects on the outcome (Y-axis). Here, the X-axis effect size comes from the LAmin volume GWAS in this manuscript, while the

Y-axis effect size comes from the Christophersen, et al, 2017 atrial fibrillation GWAS[10]. Points represent the mean effect estimates, with 95% confidence intervals for the mean.

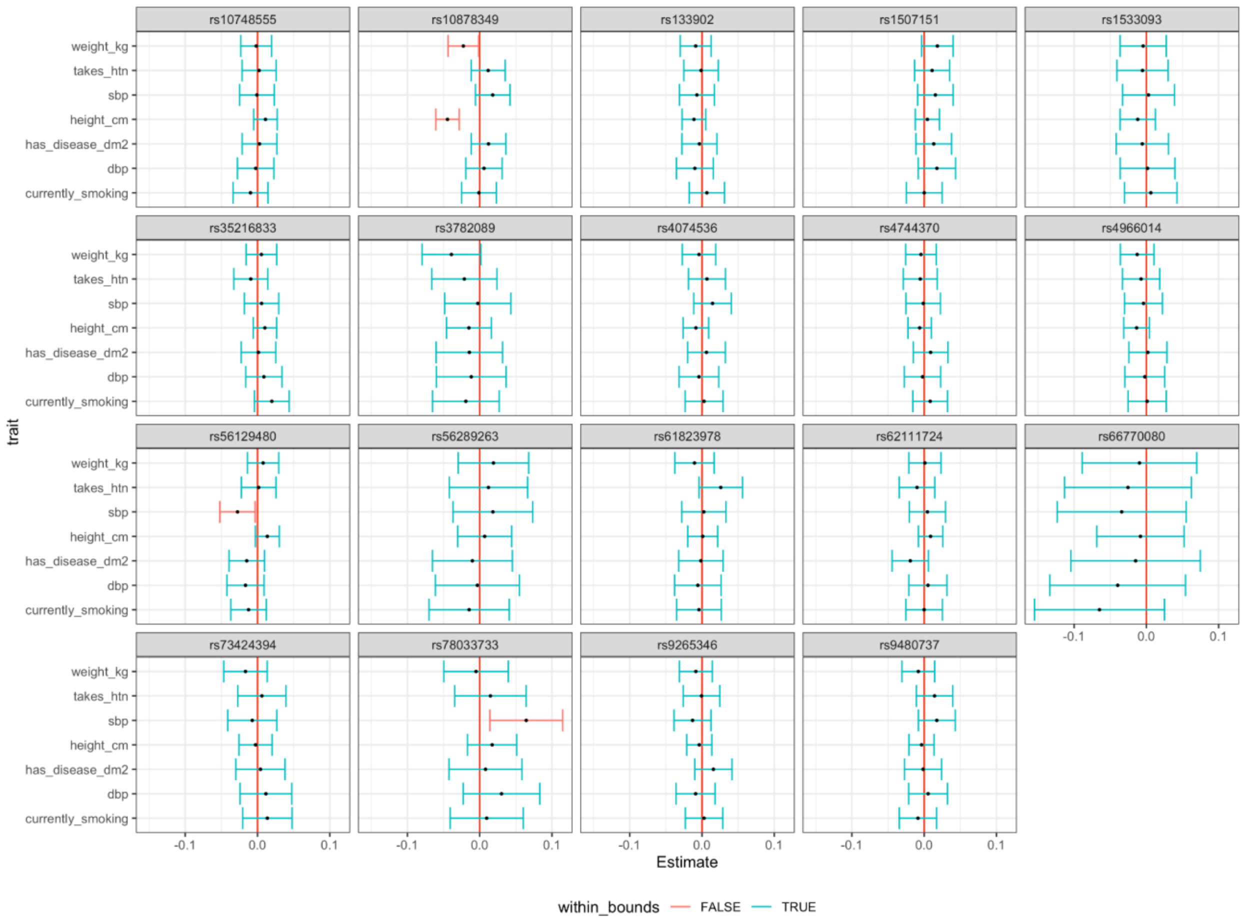

Supplementary Figure 6 - Pleiotropic associations for variants used in Mendelian randomization

Each of the 19 SNPs from the LAmin Mendelian randomization analysis was tested for association with seven phenotypes previously identified as atrial fibrillation risk factors in CHARGE-AF. For each SNP, this figure displays the mean point estimate of the effect of 1 unit change in the dosage of the non-reference allele on each trait, along with 95% confidence intervals for the mean. Traits where the association with the SNP achieves Bonferroni significance are shown in red. Three of the 19 SNPs were identified to have a significant association with at least one putative confounding factor (rs10878349 near *IRAK3*, rs56129480 near *SP3*, and rs78033733 near *MYL4*).

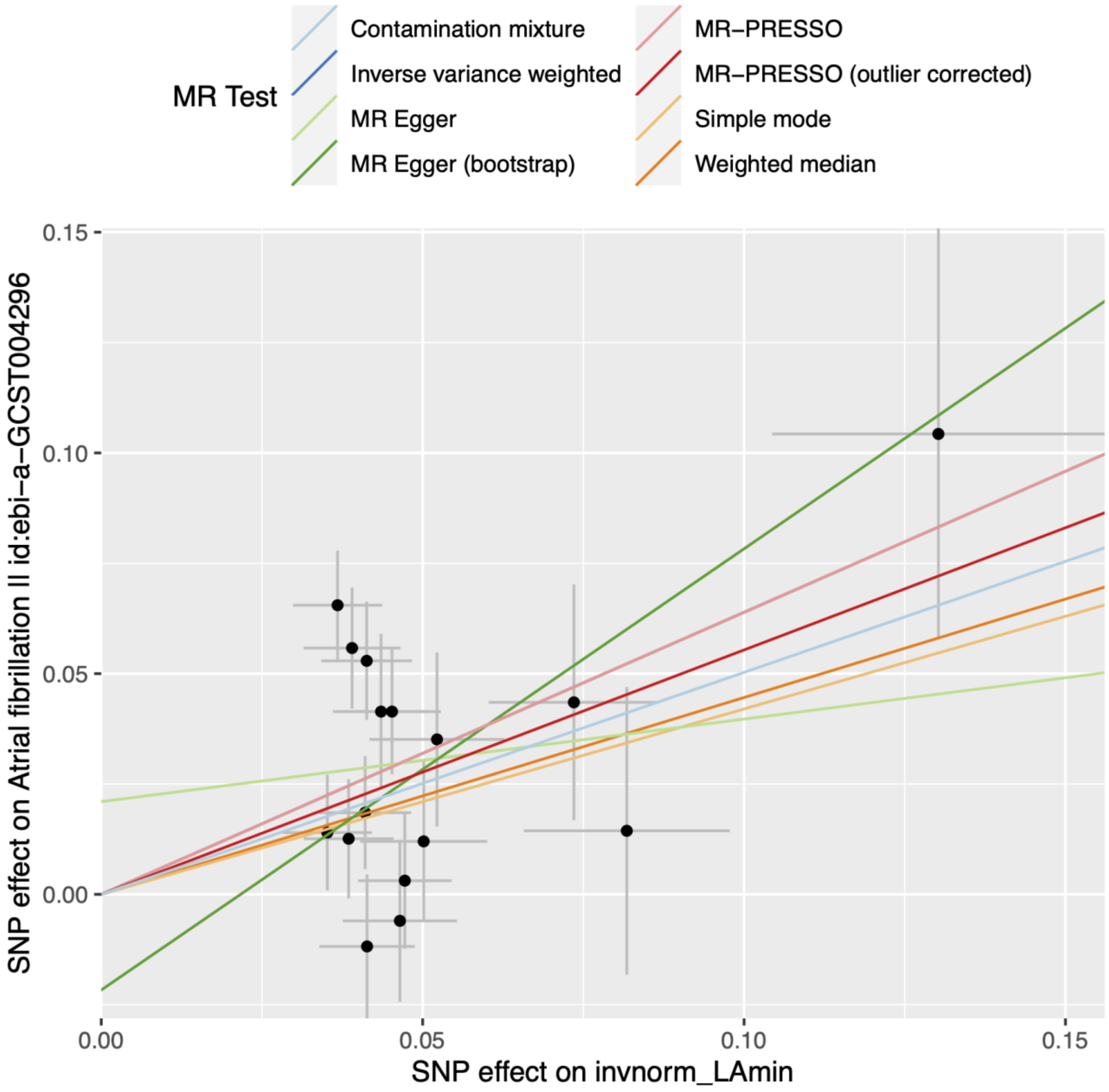

423

424 Supplementary Figure 7 - Mendelian randomization method comparison
425 plot for LAmin vs atrial fibrillation after removing 3 pleiotropic variants

426 SNP effects on the exposure (X-axis) are plotted against SNP effects on the outcome (Y-axis).
427 Here, the X-axis effect size comes from the LAmin volume GWAS in this manuscript, while the

Y-axis effect size comes from the Christophersen, et al, 2017 atrial fibrillation GWAS[10]. Points represent the mean effect estimates, with 95% confidence intervals for the mean.

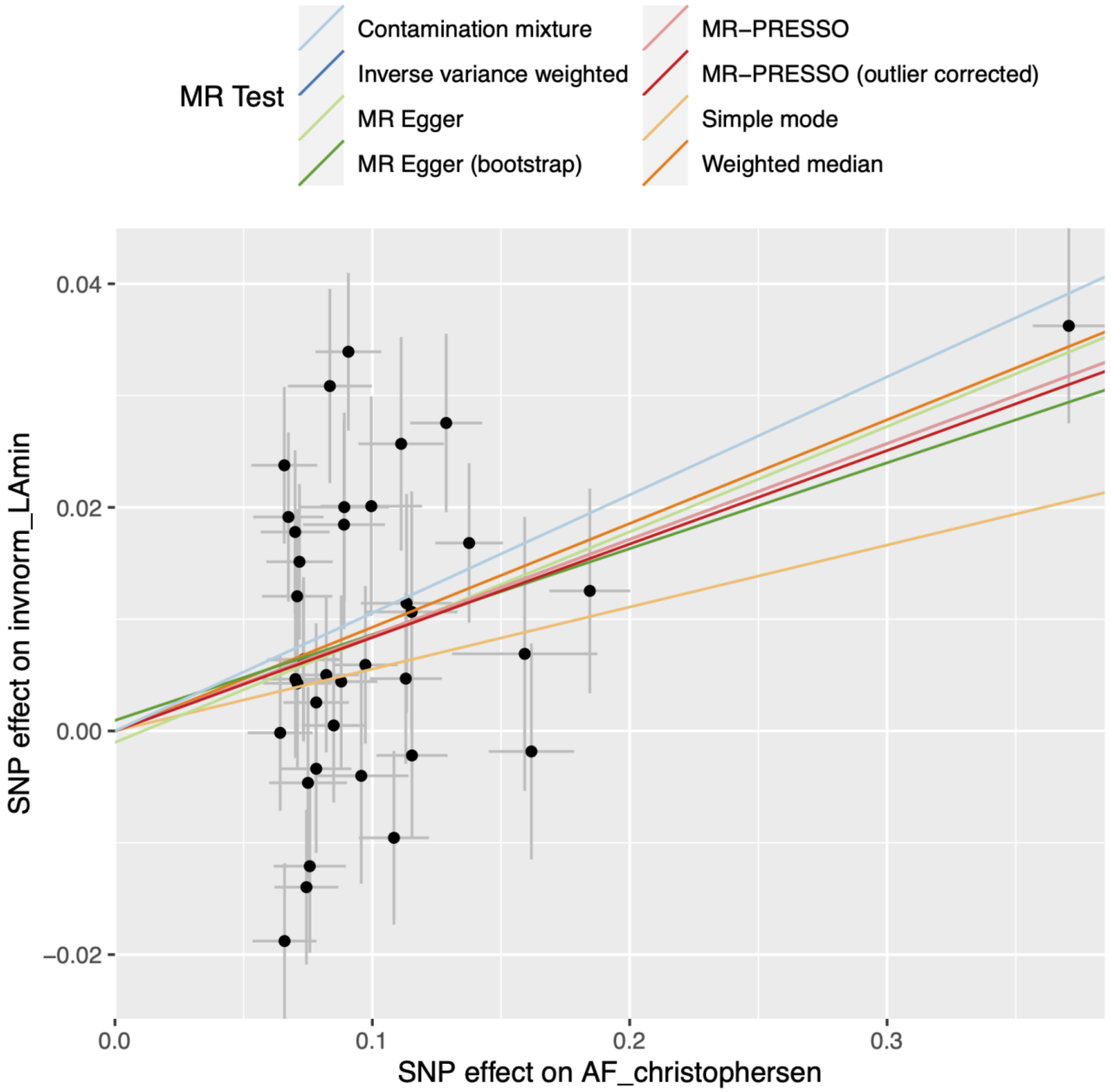

Supplementary Figure 8 - Mendelian randomization method comparison plot for atrial fibrillation vs LAmin

SNP effects on the exposure (X-axis) are plotted against SNP effects on the outcome (Y-axis). Here, the X-axis effect size comes from the Christophersen, et al, 2017 atrial fibrillation

GWAS[10], while the Y-axis effect size comes from the LAmin volume GWAS in this manuscript. Points represent the mean effect estimates, with 95% confidence intervals for the mean.

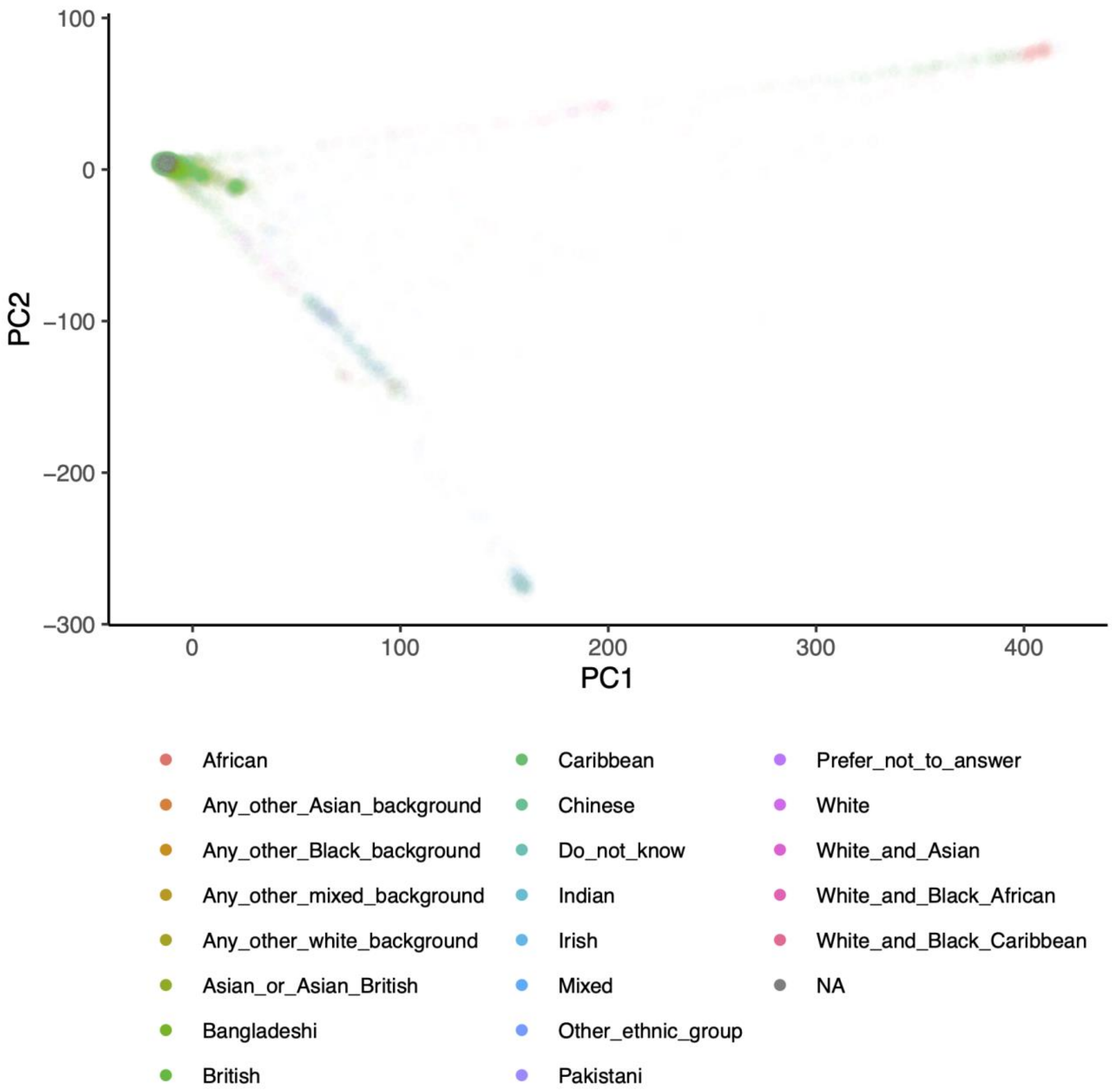

Supplementary Figure 9 - Principal components of ancestry

Principal components of ancestry for the GWAS participants, as well as participants' self-described ethnicity mapped with color.

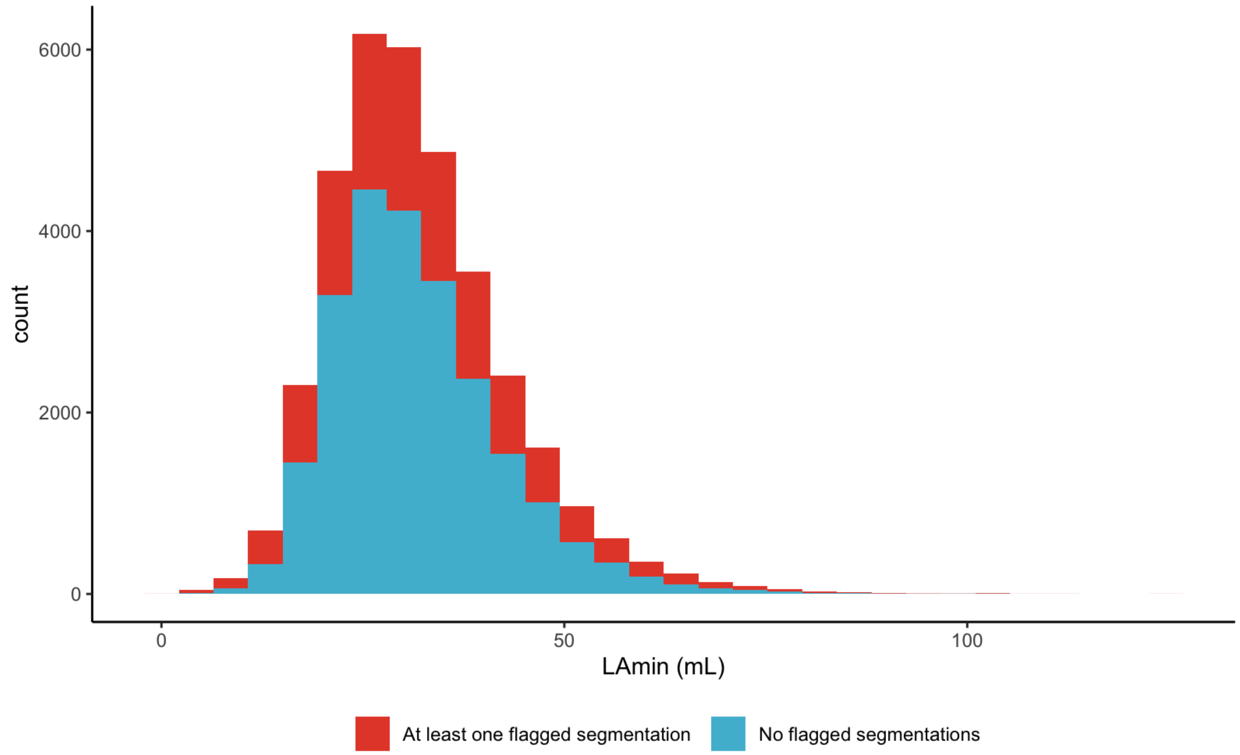

Supplementary Figure 10 - Atrial volume with or without QC flags

Histogram of distribution of LAmin volumes among participants with successful left atrial surface reconstruction. Values for those with at least one QC-flagged MRI segmentation series are colored in red, while those for participants with no flagged series are colored in turquoise. The segmentations are stacked.

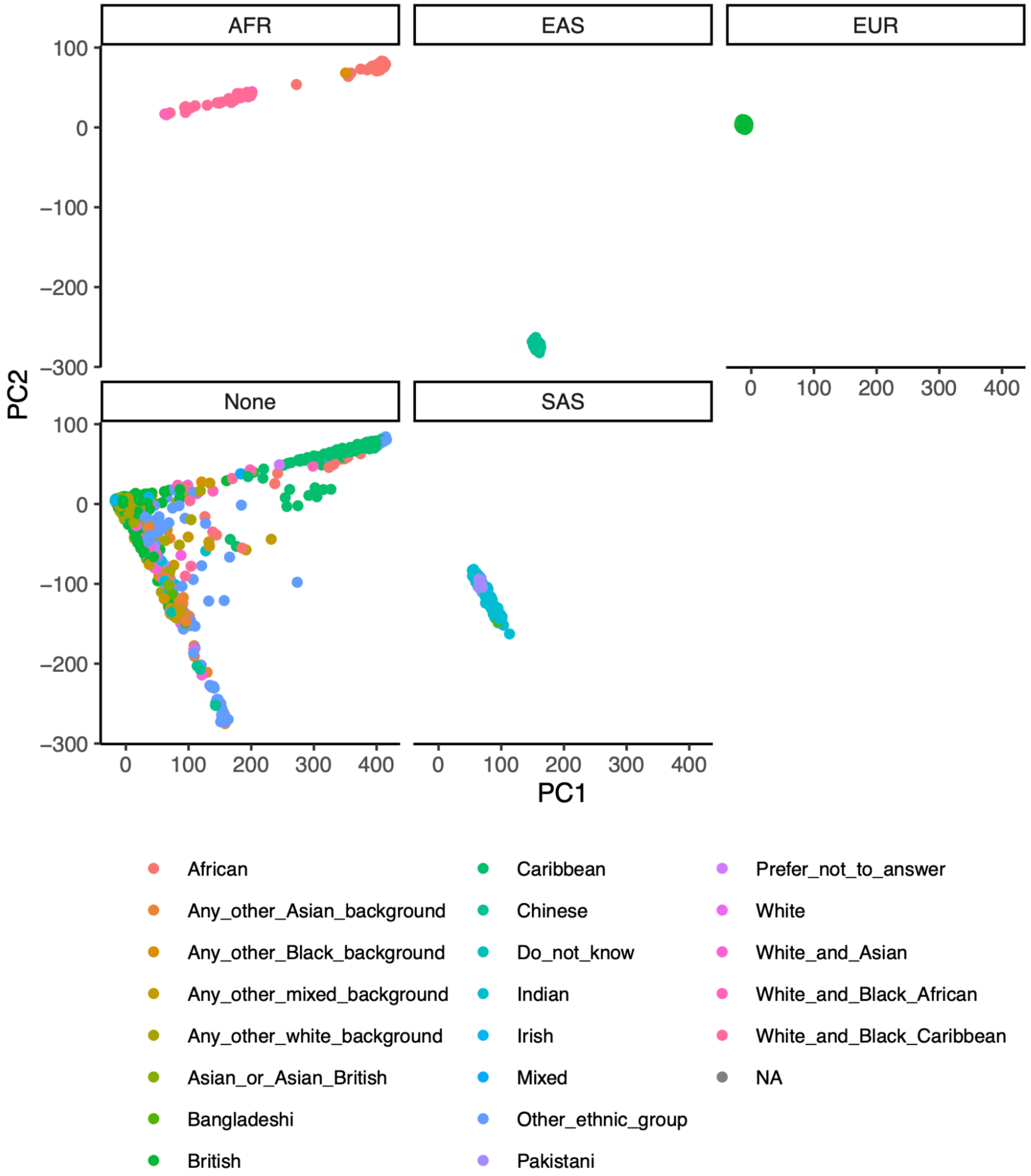

AFR
EAS
EUR
None
SAS
PC2
PC1
100
0
−100
−200
−300
0
100
200
300
400
African
Any_other_Asian_background
Any_other_Black_background
Any_other_mixed_background
Any_other_white_background
Asian_or_Asian_British
Bangladeshi
British
Caribbean
Chinese
Do_not_know
Indian
Irish
Mixed
Other_ethnic_group
Pakistani
Prefer_not_to_answer
White
White_and_Asian
White_and_Black_African
White_and_Black_Caribbean
NA

449

Supplementary Figure 11 - Principal components of ancestry by inlier group

Principal components of ancestry for the GWAS participants, as well as participants' self-described ethnicity mapped with color. Each genetic inlier group is split into its own facet. The participants that were not part of any genetic inlier group are labeled "None".

Supplementary References

1. Petersen, S. E. *et al.* UK Biobank's cardiovascular magnetic resonance protocol. *J Cardiovasc Magn Reson* **18**, (2016).
2. Petersen, S. E. *et al.* Reference ranges for cardiac structure and function using cardiovascular magnetic resonance (CMR) in Caucasians from the UK Biobank population cohort. *Journal of Cardiovascular Magnetic Resonance* **19**, 18 (2017).
3. Aschard, H., Vilhjálmsson, B. J., Joshi, A. D., Price, A. L. & Kraft, P. Adjusting for Heritable Covariates Can Bias Effect Estimates in Genome-Wide Association Studies. *Am J Hum Genet* **96**, 329–339 (2015).
4. Bellenguez, C. *et al.* A robust clustering algorithm for identifying problematic samples in genome-wide association studies. *Bioinformatics* **28**, 134–135 (2012).
5. Bycroft, C. *et al.* The UK Biobank resource with deep phenotyping and genomic data. *Nature* **562**, 203 (2018).
6. Mbatchou, J. *et al.* Computationally efficient whole-genome regression for quantitative and binary traits. *Nat Genet* **53**, 1097–1103 (2021).
7. Willer, C. J., Li, Y. & Abecasis, G. R. METAL: fast and efficient meta-analysis of genomewide association scans. *Bioinformatics* **26**, 2190–2191 (2010).
8. Sanfilippo, A. J. *et al.* Atrial enlargement as a consequence of atrial fibrillation. A prospective echocardiographic study. *Circulation* **82**, 792–797 (1990).
9. Sardana Mayank *et al.* Association of Left Atrial Function Index with Atrial Fibrillation and Cardiovascular Disease: The Framingham Offspring Study. *Journal of the American Heart Association* **7**, e008435 (2018).
10. Christophersen, I. E. *et al.* Large-scale analyses of common and rare variants identify 12 new loci associated with atrial fibrillation. *Nat Genet* **49**, 946–952 (2017).

FinnGen Consortium

Aarno Palotie[8,19,20], Mark Daly[21], Bridget Riley-Gills[22], Howard Jacob[22], Dirk Paul[23], Athena Matakidou[23], Adam Platt[23], Heiko Runz[24], Sally John[24], George Okafo[25], Nathan Lawless[25], Heli Salminen-Mankonen[25], Robert Plenge[26], Joseph Maranville[26], Mark McCarthy[27], Julie Hunkapiller[27], Margaret G. Ehm[28], Kirsi Auro[29], Simonne Longerich[30], Caroline Fox[30], Anders Mälarstig[31], Katherine Klinger[32], Deepak Raipal[32], Eric Green[33], Robert Graham[33], Robert Yang[34], Chris O´Donnell[35], Tomi P. Mäkelä[36], Jaakko Kaprio[37], Petri Virolainen[38], Antti Hakanen[38], Terhi Kilpi[39], Markus Perola[39], Jukka Partanen[40], Anne Pitkäranta[41], Taneli Raivio[41], Raisa Serpi[42], Tarja Laitinen[43], Veli-Matti Kosma[44], Jari Laukkanen[45], Marco Hautalahti[46], Outi Tuovila[47], Raimo Pakkanen[47], Jeffrey Waring[22], Bridget Riley-Gillis[22], Fedik Rahimov[22], Ioanna Tachmazidou[23], Chia-Yen Chen[24], Heiko Runz[24], Zhihao Ding[25], Marc Jung[25], Shameek Biswas[26], Rion Pendergrass[27], Julie Hunkapiller[27], Margaret G. Ehm[28], David Pulford[48], Neha Raghavan[30], Adriana Huertas-Vazquez[30], Jae-Hoon Sul[30], Anders Mälarstig[31], Xinli Hu[31], Katherine Klinger[32], Robert Graham[33], Eric Green[33], Sahar Mozaffari[33], Dawn Waterworth[49], Nicole Renaud[35], Ma´en Obeidat[35], Samuli Ripatti[37], Johanna Schleutker[50], Markus Perola[39], Mikko Arvas[40], Olli Carpén[41], Reetta Hinttala[42], Johannes Kettunen[42], Arto Mannermaa[44], Katriina Aalto-Setälä[51], Mika Kähönen[43], Jari Laukkanen[45], Johanna Mäkelä[46], Reetta Kälviäinen[52], Valtteri Julkunen[52], Hilkka Soininen[52], Anne Remes[53], Mikko Hiltunen[54], Jukka Peltola[55], Minna Raivio[56], Pentti Tienari[56], Juha Rinne[57], Roosa Kallionpää[57], Juulia Partanen[58], Ali Abbasi[22], Adam Ziemann[22], Nizar Smaoui[22], Anne Lehtonen[22], Susan Eaton[24], Heiko Runz[24], Sanni Lahdenperä[24], Shameek Biswas[26], Julie Hunkapiller[27], Natalie Bowers[27], Edmond Teng[27], Rion Pendergrass[27], Fanli Xu[59], David Pulford[48], Kirsi Auro[29], Laura Addis[59], John Eicher[59], Qingqin S Li[60], Karen He[49], Ekaterina Khramtsova[49], Neha Raghavan[30], Martti Färkkilä[56], Jukka Koskela[56], Sampsa Pikkarainen[56], Airi Jussila[55], Katri Kaukinen[55], Timo Blomster[53], Mikko Kiviniemi[52], Markku Voutilainen[57], Mark Daly[61], Ali Abbasi[22], Jeffrey Waring[22], Nizar Smaoui[22], Fedik Rahimov[22], Anne Lehtonen[22], Tim Lu[27], Natalie Bowers[27], Rion Pendergrass[27], Linda

McCarthy[59], Amy Hart[49], Meijian Guan[49], Jason Miller[30], Kirsi Kalpala[31], Melissa Miller[31], Xinli Hu[31], Kari Eklund[56], Antti Palomäki[57], Pia Isomäki[55], Laura Pirilä[57], Oili Kaipiainen-Seppänen[52], Johanna Huhtakangas[53], Nina Mars[37], Ali Abbasi[22], Jeffrey Waring[22], Fedik Rahimov[22], Apinya Lertratanakul[22], Nizar Smaoui[22], Anne Lehtonen[22], Marla Hochfeld[26], Natalie Bowers[27], Rion Pendergrass[27], Jorge Esparza Gordillo[59], Kirsi Auro[29], Dawn Waterworth[49], Fabiana Farias[30], Kirsi Kalpala[31], Nan Bing[31], Xinli Hu[31], Tarja Laitinen[55], Margit Pelkonen[52], Paula Kauppi[56], Hannu Kankaanranta[62], Terttu Harju[53], Riitta Lahesmaa[57], Nizar Smaoui[22], Glenda Lassi[23], Susan Eaton[24], Hubert Chen[27], Rion Pendergrass[27], Natalie Bowers[27], Joanna Betts[59], Kirsi Auro[29], Rajashree Mishra[59], Majd Mouded[63], Debby Ngo[63], Teemu Niiranen[64], Felix Vaura[64], Veikko Salomaa[64], Kaj Metsärinne[57], Jenni Aittokallio[57], Mika Kähönen[55], Jussi Hernesniemi[55], Daniel Gordin[56], Juha Sinisalo[56], Marja-Riitta Taskinen[56], Tiinamaija Tuomi[56], Timo Hiltunen[56], Jari Laukkanen[65], Amanda Elliott[66], Mary Pat Reeve[37], Sanni Ruotsalainen[37], Benjamin Challis[23], Dirk Paul[23], Julie Hunkapiller[27], Natalie Bowers[27], Rion Pendergrass[27], Audrey Chu[59], Kirsi Auro[29], Dermot Reilly[67], Mike Mendelson[68], Jaakko Parkkinen[31], Melissa Miller[31], Tuomo Meretoja[56], Heikki Joensuu[56], Olli Carpén[56], Johanna Mattson[56], Eveliina Salminen[56], Annika Auranen[69], Peeter Karihtala[53], Päivi Auvinen[52], Klaus Elenius[57], Johanna Schleutker[57], Esa Pitkänen[37], Nina Mars[37], Mark Daly[21], Relja Popovic[22], Jeffrey Waring[22], Bridget Riley-Gillis[22], Anne Lehtonen[22], Jennifer Schutzman[27], Julie Hunkapiller[27], Natalie Bowers[27], Rion Pendergrass[27], Diptee Kulkarni[59], Kirsi Auro[29], Alessandro Porello[49], Andrey Loboda[30], Heli Lehtonen[31], Stefan McDonough[31], Sauli Vuoti[70], Kai Kaarniranta[52], Joni A Turunen[71], Terhi Ollila[56], Hannu Uusitalo[55], Juha Karjalainen[37], Esa Pitkänen[37], Mengzhen Liu[22], Heiko Runz[24], Stephanie Loomis[24], Erich Strauss[27], Natalie Bowers[27], Hao Chen[27], Rion Pendergrass[27], Kaisa Tasanen[53], Laura Huilaja[53], Katariina Hannula-Jouppi[56], Teea Salmi[55], Sirkku Peltonen[57], Leena Koulu[57], Nizar Smaoui[22], Fedik Rahimov[22], Anne Lehtonen[22], David Choy[27], Rion Pendergrass[27], Dawn Waterworth[49], Kirsi Kalpala[31], Ying Wu[31], Pirkko Pussinen[56], Aino Salminen[56], Tuula Salo[56], David Rice[56], Pekka Nieminen[56], Ulla Palotie[56], Maria Siponen[52], Liisa Suominen[52], Päivi

Mäntylä[52], Ulvi Gursoy[57], Vuokko Anttonen[53], Kirsi Sipilä[72], Rion Pendergrass[27], Hannele Laivuori[37], Venla Kurra[55], Laura Kotaniemi-Talonen[55], Oskari Heikinheimo[56], Ilkka Kalliala[56], Lauri Aaltonen[56], Varpu Jokimaa[57], Johannes Kettunen[53], Marja Vääräsmäki[53], Outi Uimari[53], Laure Morin-Papunen[53], Maarit Niinimäki[53], Terhi Piltonen[53], Katja Kivinen[37], Elisabeth Widen[37], Taru Tukiainen[37], Mary Pat Reeve[37], Mark Daly[21], Niko Välimäki[73], Eija Laakkonen[74], Jaakko Tyrmi[75], Heidi Silven[76], Eeva Sliz[76], Riikka Arffman[76], Susanna Savukoski[76], Triin Laisk[77], Natalia Pujol[77], Mengzhen Liu[22], Bridget Riley-Gillis[22], Rion Pendergrass[27], Janet Kumar[28], Kirsi Auro[29], Iiris Hovatta[78], Chia-Yen Chen[24], Erkki Isometsä[56], Hanna Ollila[37], Jaana Suvisaari[64], Thomas Damm Als[79], Antti Mäkitie[80], Argyro Bizaki-Vallaskangas[55], Sanna Toppila-Salmi[78], Tytti Willberg[57], Elmo Saarentaus[37], Antti Aarnisalo[56], Eveliina Salminen[56], Elisa Rahikkala[53], Johannes Kettunen[53], Kristiina Aittomäki[81], Fredrik Ãberg[82], Mitja Kurki[83], Samuli Ripatti[37], Mark Daly[61], Juha Karjalainen[37], Aki Havulinna[84], Juha Mehtonen[37], Priit Palta[37], Shabbeer Hassan[37], Pietro Della Briotta Parolo[37], Wei Zhou[85], Mutaamba Maasha[85], Shabbeer Hassan[37], Susanna Lemmelä[37], Manuel Rivas[86], Mari E. Niemi[37], Aarno Palotie[37], Aoxing Liu[37], Arto Lehisto[37], Andrea Ganna[37], Vincent Llorens[37], Hannele Laivuori[37], Taru Tukiainen[37], Mary Pat Reeve[37], Henrike Heyne[37], Nina Mars[37], Joel Rämö[37], Elmo Saarentaus[37], Hanna Ollila[37], Rodos Rodosthenous[37], Satu Strausz[37], Tuula Palotie[87], Kimmo Palin[73], Javier Garcia-Tabuenca[88], Harri Siirtola[88], Tuomo Kiiskinen[37], Jiwoo Lee[83], Kristin Tsuo[83], Amanda Elliott[66], Kati Kristiansson[39], Mikko Arvas[40], Kati Hyvärinen[89], Jarmo Ritari[89], Olli Carpén[41], Johannes Kettunen[42], Katri Pylkäs[76], Eeva Sliz[76], Minna Karjalainen[76], Tuomo Mantere[42], Eeva Kangasniemi[43], Sami Heikkinen[54], Arto Mannermaa[44], Eija Laakkonen[74], Nina Pitkänen[38], Samuel Lessard[32], Clément Chatelain[32], Perttu Terho[38], Sirpa Soini[39], Jukka Partanen[40], Eero Punkka[41], Raisa Serpi[42], Sanna Siltanen[43], Veli-Matti Kosma[44], Teijo Kuopio[45], Anu Jalanko[37], Huei-Yi Shen[37], Risto Kajanne[37], Mervi Aavikko[37], Henna Palin[43], Malla-Maria Linna[41], Mitja Kurki[83], Juha Karjalainen[37], Pietro Della Briotta Parolo[37], Arto Lehisto[37], Juha Mehtonen[37], Wei Zhou[85], Masahiro Kanai[85], Mutaamba Maasha[85], Hannele Laivuori[37], Aki Havulinna[84], Susanna

Lemmelä[37], Tuomo Kiiskinen[37], L. Elisa Lahtela[37], Mari Kaunisto[37], Elina Kilpeläinen[37], Timo P. Sipilä[37], Oluwaseun Alexander Dada[37], Awaisa Ghazal[37], Anastasia Kytölä[37], Rigbe Weldatsadik[37], Sanni Ruotsalainen[37], Kati Donner[37], Timo P. Sipilä[37], Anu Loukola[41], Päivi Laiho[39], Tuuli Sistonen[39], Essi Kaiharju[39], Markku Laukkanen[39], Elina Järvensivu[39], Sini Lähteenmäki[39], Lotta Männikkö[39], Regis Wong[39], Auli Toivola[39], Minna Brunfeldt[39], Hannele Mattsson[39], Kati Kristiansson[39], Susanna Lemmelä[37], Sami Koskelainen[39], Tero Hiekkalinna[39], Teemu Paajanen[39], Priit Palta[37], Kalle Pärn[37], Mart Kals[37], Shuang Luo[37], Tarja Laitinen[55], Mary Pat Reeve[37], Shanmukha Sampath Padmanabhuni[37], Marianna Niemi[88], Harri Siirtola[88], Javier Gracia-Tabuenca[88], Mika Helminen[88], Tiina Luukkaala[88], Iida Vähätalo[88], Jyrki Pitkänen[37], Marco Hautalahti[90], Johanna Mäkelä[90], Sarah Smith[90], Tom Southerington[90]

8 Institute for Molecular Medicine Finland (FIMM), Helsinki Institute of Life Science (HiLIFE), University of Helsinki

19 Analytic and Translational Genetics Unit, Massachusetts General Hospital and Harvard Medical School

20 Stanley Center for Psychiatric Research, Broad Institute of MIT and Harvard

21 Institute for Molecular Medicine Finland (FIMM), HiLIFE, University of Helsinki, Helsinki, Finland; Broad Institute of MIT and Harvard; Massachusetts General Hospital

22 Abbvie, Chicago, IL, United States

23 Astra Zeneca, Cambridge, United Kingdom

24 Biogen, Cambridge, MA, United States

25 Boehringer Ingelheim, Ingelheim am Rhein, Germany

26 Bristol Myers Squibb, New York, NY, United States

27 Genentech, San Francisco, CA, United States

28 GlaxoSmithKline, Collegeville, PA, United States

29 GlaxoSmithKline, Espoo, Finland

30 Merck, Kenilworth, NJ, United States

31 Pfizer, New York, NY, United States

32 Translational Sciences, Sanofi R&D, Framingham, MA, USA

33 Maze Therapeutics, San Francisco, CA, United States

34 Janssen Biotech, Beerse, Belgium

35 Novartis Institutes for BioMedical Research, Cambridge, MA, United States

36 HiLIFE, University of Helsinki, Finland, Finland

37 Institute for Molecular Medicine Finland (FIMM), HiLIFE, University of Helsinki, Helsinki, Finland

38 Auria Biobank / University of Turku / Hospital District of Southwest Finland, Turku, Finland

39 THL Biobank / Finnish Institute for Health and Welfare (THL), Helsinki, Finland

40 Finnish Red Cross Blood Service / Finnish Hematology Registry and Clinical Biobank, Helsinki, Finland

41 Helsinki Biobank / Helsinki University and Hospital District of Helsinki and Uusimaa, Helsinki

42 Northern Finland Biobank Borealis / University of Oulu / Northern Ostrobothnia Hospital District, Oulu, Finland

43 Finnish Clinical Biobank Tampere / University of Tampere / Pirkanmaa Hospital District, Tampere, Finland

44 Biobank of Eastern Finland / University of Eastern Finland / Northern Savo Hospital District, Kuopio, Finland

45 Central Finland Biobank / University of Jyväskylä / Central Finland Health Care District, Jyväskylä, Finland

46 FINBB - Finnish biobank cooperative

47 Business Finland, Helsinki, Finland

48 GlaxoSmithKline, Stevenage, United Kingdom

49 Janssen Research & Development, LLC, Spring House, PA, United States

50 Auria Biobank / Univ. of Turku / Hospital District of Southwest Finland, Turku, Finland

51 Faculty of Medicine and Health Technology, Tampere University, Tampere, Finland

52 Northern Savo Hospital District, Kuopio, Finland

53 Northern Ostrobothnia Hospital District, Oulu, Finland

54 University of Eastern Finland, Kuopio, Finland

55 Pirkanmaa Hospital District, Tampere, Finland

56 Hospital District of Helsinki and Uusimaa, Helsinki, Finland

57 Hospital District of Southwest Finland, Turku, Finland

58 Institute for Molecular Medicine Finland, HiLIFE, University of Helsinki, Finland

59 GlaxoSmithKline, Brentford, United Kingdom

60 Janssen Research & Development, LLC, Titusville, NJ 08560, United States

61 Institute for Molecular Medicine, Finland (FIMM), HiLIFE, University of Helsinki, Helsinki, Finland; Broad Institute of MIT and Harvard; Massachusetts General Hospital

62 University of Gothenburg, Gothenburg, Sweden/ Seinäjoki Central Hospital, Seinäjoki, Finland/ Tampere University, Tampere, Finland

63 Novartis, Basel, Switzerland

64 Finnish Institute for Health and Welfare (THL), Helsinki, Finland

65 Central Finland Health Care District, Jyväskylä, Finland

66 Institute for Molecular Medicine Finland (FIMM), HiLIFE, University of Helsinki, Helsinki, Finland; Broad Institute, Cambridge, MA, USA and Massachusetts General Hospital, Boston, MA, USA

67 Janssen Research & Development, LLC, Boston, MA, United States

68 Novartis, Boston, MA, United States

69 Pirkanmaa Hospital District , Tampere, Finland

70 Janssen-Cilag Oy, Espoo, Finland

71 Helsinki University Hospital and University of Helsinki, Helsinki, Finland; Eye Genetics

Group, Folkhälsan Research Center, Helsinki, Finland

72 Research Unit of Oral Health Sciences Faculty of Medicine, University of Oulu, Oulu, Finland; Medical Research Center, Oulu, Oulu University Hospital and University of Oulu, Oulu, Finland

73 University of Helsinki, Helsinki, Finland

74 University of Jyväskylä, Jyväskylä, Finland

75 University of Oulu, Oulu, Finland / University of Tampere, Tampere, Finland

76 University of Oulu, Oulu, Finland

77 Estonian biobank, Tartu, Estonia

78 University of Helsinki, Finland

79 Aarhus University, Denmark

80 Department of Otorhinolaryngology - Head and Neck Surgery, University of Helsinki and Helsinki University Hospital, Helsinki, Finland

81 Department of Medical Genetics, Helsinki University Central Hospital, Helsinki, Finland

82 Transplantation and Liver Surgery Clinic, Helsinki University Hospital, Helsinki University, Helsinki, Finland

83 Institute for Molecular Medicine Finland (FIMM), HiLIFE, University of Helsinki, Helsinki, Finland; Broad Institute, Cambridge, MA, United States

84 Institute for Molecular Medicine Finland (FIMM), HiLIFE, University of Helsinki, Helsinki, Finland; Finnish Institute for Health and Welfare (THL), Helsinki, Finland

85 Broad Institute, Cambridge, MA, United States

86 University of Stanford, Stanford, CA, United States

87 University of Helsinki and Hospital District of Helsinki and Uusimaa, Helsinki, Finland

88 University of Tampere, Tampere, Finland

89 Finnish Red Cross Blood Service, Helsinki, Finland

90 Finnish Biobank Cooperative - FINBB
