# Supplementary material for: Deep learning of left atrial structure and function provides link to atrial fibrillation risk
Source: Nat Commun. 2024 May 21;15:4304. doi: 10.1038/s41467-024-48229-w (PMC11109224; doi:10.1038/s41467-024-48229-w)
Supplement: Supplementary file 3 — Description of Additional Supplementary Files [file 41467_2024_48229_MOESM3_ESM.docx]

**Title: Supplementary Data 1**

**Description**: Continuous PheWAS. IndepVariable: Independent variable. OutcomeTrait: Dependent variable. TraitFieldID: UK Biobank FieldID value, where relevant. N_With_Variable: Number of individuals with independent variable. N_With_Trait: Number of individuals with outcome trait. N_Modeled: Number of individuals having both the independent variable and the outcome trait, therefore able to be modeled. Beta: Effect size. SE: Standard error. P: Two-tailed P-value. AbsZ: Absolute value of (Beta/SE). Issue: If the modeling procedure had convergence issues, they are printed here. YourVal: Always ‘dependent’. Formula: The R formula for the test.

**Title: Supplementary Data 2**

**Description**: Hardy-Weinberg P values and GWAS sensitivity analysis based on genetic identity groupings. Chromosomal positions are in GRCh37 coordinates. ALLELE1 is the effect allele. HWE_Exact_P: The two-tailed Hardy-Weinberg P values at the locus. P_MAIN: P-value in the main GWAS. P_EUR: P-value of the EUR-specific GWAS. P_RANDOMDROP: P-value in the sensitivity analysis GWAS in which random individuals of equal number were excluded from the analysis rather than non-EUR individuals. P_META: Association P-value from METAL for the meta-analysis of EUR, AFR, SAS, and EAS-specific GWAS.

**Title: Supplementary Data 3**

**Description**: GWAS lead SNPs for the primary and LV-indexed analyses. Lead SNPs for the primary LA phenotypes, the body surface area-indexed LA phenotypes, and the left ventricular end diastolic volume-indexed LA phenotypes are depicted.

**Title: Supplementary Data 4**

**Description**: Mendelian randomization sensitivity analysis and pleiotropy. The ‘analysis’ column distinguishes the primary Mendelian randomization (MR) analysis from the sensitivity analysis that excluded pleiotropic SNPs. The intercept and intercept P value (tested against a null hypothesis of an intercept of 0) are shown for MR Egger analyses.

**Title: Supplementary Data 5**

**Description**: Mendelian randomization sensitivity analysis using P<5E-08 instruments. Mendelian randomization (MR) results for analyses using only genome-wide significant instruments are shown.

**Title: Supplementary Data 6**

**Description**: LA PRS on other disease risk, as well as PRS sensitivity analyses using lead SNP PRS instead of PRScs. The left atrial polygenic scores were tested for association with diseases in the UK Biobank. Sensitivity analyses include polygenic scores defined using lead SNPs instead of ~1.1 million-SNP Bayesian genome-wide panels, thresholded analysis comparing the top 5% to the remaining 95%, and analyses conducted within the EUR inlier group.

**Title: Supplementary Data 7**

**Description**: FinnGen disease definitions.

**Title: Supplementary Data 8**

**Description**: All of Us polygenic score analysis. The body surface area-indexed LAmin polygenic score was tested in All of Us for association with atrial fibrillation, heart failure, and ischemic stroke, both in all-comers and in the EUR subset.

**Title: Supplementary Data 9**

**Description**: Disease definitions in UK Biobank.

**Title: Supplementary Data 10**

**Description**: Antihypertensive medication definition in UK Biobank.

**Title: Supplementary Data 11**

**Description**: GWAS sensitivity analysis without the cardiac motion filter. Results are shown for both LAmin and body surface area-indexed LAmin.
