# Supplementary material for: Deep learning of left atrial structure and function provides link to atrial fibrillation risk
Source: Nat Commun. 2024 May 21;15:4304. doi: 10.1038/s41467-024-48229-w (PMC11109224; doi:10.1038/s41467-024-48229-w)
Supplement: Supplementary file 15 — Reporting Summary [file 41467_2024_48229_MOESM15_ESM.pdf]

nature portfolio

Corresponding author(s): James Pirruccello

Last updated by author(s): 3/12/2024

Reporting Summary

Nature Portfolio wishes to improve the reproducibility of the work that we publish. This form provides structure for consistency and transparency in reporting. For further information on Nature Portfolio policies, see our Editorial Policies and the Editorial Policy Checklist.

Statistics

For all statistical analyses, confirm that the following items are present in the figure legend, table legend, main text, or Methods section.

n/a	Confirmed	
☐	☒	The exact sample size (*n*) for each experimental group/condition, given as a discrete number and unit of measurement
☐	☒	A statement on whether measurements were taken from distinct samples or whether the same sample was measured repeatedly
☐	☒	The statistical test(s) used AND whether they are one- or two-sided<br>*Only common tests should be described solely by name; describe more complex techniques in the Methods section.*
☐	☒	A description of all covariates tested
☐	☒	A description of any assumptions or corrections, such as tests of normality and adjustment for multiple comparisons
☐	☒	A full description of the statistical parameters including central tendency (e.g. means) or other basic estimates (e.g. regression coefficient) AND variation (e.g. standard deviation) or associated estimates of uncertainty (e.g. confidence intervals)
☐	☒	For null hypothesis testing, the test statistic (e.g. *F*, *t*, *r*) with confidence intervals, effect sizes, degrees of freedom and *P* value noted<br>*Give P values as exact values whenever suitable.*
☒	☐	For Bayesian analysis, information on the choice of priors and Markov chain Monte Carlo settings
☒	☐	For hierarchical and complex designs, identification of the appropriate level for tests and full reporting of outcomes
☐	☒	Estimates of effect sizes (e.g. Cohen's *d*, Pearson's *r*), indicating how they were calculated

Our web collection on statistics for biologists contains articles on many of the points above.

Software and code

Policy information about availability of computer code

Data collection	Cardiac MRI images were manually annotated with traceoverlay v0.1.0 (https://zenodo.org/records/10811511).
Data analysis	Deep learning models were trained with fastai v1.0.6. Detection of abnormal contraction was performed using models trained with FastAI version 2.2.5 and the TimeSeries AI library version 0.2.15. 3D volumes were reconstructed using the Screened Poisson Surface Reconstruction C++ Library v6.13 as implemented at https://github.com/broadinstitute/ml4h/blob (https://zenodo.org/records/10811233)<br><br>Genome-wide association studies were conducted with BOLT-LMM v2.3.4. Plink-1.9 was used to clump lead variants; ldsc v1.0.0 was used for LD score regression and ldsc v1.0.1 was used to perform cross-trait LD score regression. TwoSampleMR v0.5.7 was used for Mendelian randomization.

For manuscripts utilizing custom algorithms or software that are central to the research but not yet described in published literature, software must be made available to editors and reviewers. We strongly encourage code deposition in a community repository (e.g. GitHub). See the Nature Portfolio guidelines for submitting code & software for further information.

Data

Policy information about availability of data

All manuscripts must include a data availability statement. This statement should provide the following information, where applicable:

- Accession codes, unique identifiers, or web links for publicly available datasets
- A description of any restrictions on data availability
- For clinical datasets or third party data, please ensure that the statement adheres to our policy

GWAS summary statistics are available in the GWAS Catalog under accession #GCP000842.

UK Biobank data are made available to researchers from research institutions with genuine research inquiries, following IRB and UK Biobank approval. GWAS summary statistics and polygenic score weights will be available upon publication at the Broad Institute Cardiovascular Disease Knowledge Portal (http://www.broadcvdi.org). LA measurements have been returned to the UK Biobank for use by any approved researcher. All of Us data are available for analysis to qualified researchers on the All of Us research platform. FinnGen Freeze 9 GWAS summary statistics are available at https://www.finngen.fi/en/access_results. All other data are contained within the article and its supplementary information.

Research involving human participants, their data, or biological material

Policy information about studies with human participants or human data. See also policy information about sex, gender (identity/presentation), and sexual orientation and race, ethnicity and racism.

Reporting on sex and gender	The data come from UK Biobank, which provides information about sex but not gender. Therefore, analyses such as GWAS and cardiovascular risk were analyzed with respect to sex, but not gender.
Reporting on race, ethnicity, or other socially relevant groupings	In the primary analysis, ethnicity/race were not considered - all participants were included regardless of race, ethnicity, or other group. In secondary analyses, genetic inlier groups that were seeded by self-reported ethnicity but ultimately defined by principal components of ancestry were constructed to address questions about the possibility of population stratification. It is important to note that race and ethnicity are not genetically determined.
Population characteristics	UK Biobank participants were recruited between the ages of 40-70 and have a slight predominance of women over men compared to the UK population baseline. The most common genetic identity group represented is one similar to that of Europeans, while there are participants with diverse genetic identities including those similar to individuals in East Asia, South Asia, and Africa.
Recruitment	Participants in the UK Biobank were recruited via a study protocol that has been described extensively and with subsequent biases that are most prominent for behavioral traits, which are not analyzed here. A relevant manuscript describing these can be found at https://pubmed.ncbi.nlm.nih.gov/37106081/
Ethics oversight	Access was provided under application #7089 and approved by the Partners HealthCare institutional review board (protocol 2019P003144). Analysis of All of Us was considered exempt by the UCSF IRB (#22-37715).

Note that full information on the approval of the study protocol must also be provided in the manuscript.

Field-specific reporting

Please select the one below that is the best fit for your research. If you are not sure, read the appropriate sections before making your selection.

☒ Life sciences ☐ Behavioural & social sciences ☐ Ecological, evolutionary & environmental sciences

For a reference copy of the document with all sections, see nature.com/documents/nr-reporting-summary-flat.pdf

Life sciences study design

All studies must disclose on these points even when the disclosure is negative.

Sample size	The sample size was determined based on the use of all available MRI data from UK Biobank at the time of the study. 40,558 participants had MRI measurements that passed QC and 35,049 participated in the genome-wide association study for left atrial structure and function.
Data exclusions	Data were excluded due to abnormal atrial contraction (to avoid reverse causation with atrial fibrillation), due to pre-existing cardiovascular disease, or due to missing genetic data or failing genetic QC (sample QC missing rate >= 2%, sex chromosome aneuploidy, or outlier status for heterozygosity)
Replication	External validation of the polygenic score was performed in FinnGen and All of Us, which confirmed the primary analysis with atrial fibrillation found in UK Biobank as well as secondary analyses with heart failure.
Randomization	No randomization was performed as this was not a prospective controlled study. Rather, covariates such as age, sex, and genetic principal components were adjusted for in the statistical model.
Blinding	Blinding was not performed in this retrospective analysis, because no intervention was performed and no interaction with any participants

occurred. Because the traits were quantitative (atrial size and function), there was also no case/control status to be blind to during annotation or during the primary analyses.

Reporting for specific materials, systems and methods

We require information from authors about some types of materials, experimental systems and methods used in many studies. Here, indicate whether each material, system or method listed is relevant to your study. If you are not sure if a list item applies to your research, read the appropriate section before selecting a response.

Materials & experimental systems

n/a	Involved in the study
☒	☐ Antibodies
☒	☐ Eukaryotic cell lines
☒	☐ Palaeontology and archaeology
☒	☐ Animals and other organisms
☒	☐ Clinical data
☒	☐ Dual use research of concern
☒	☐ Plants

Methods

n/a	Involved in the study
☒	☐ ChIP-seq
☒	☐ Flow cytometry
☒	☐ MRI-based neuroimaging

Plants

Seed stocks

Report on the source of all seed stocks or other plant material used. If applicable, state the seed stock centre and catalogue number. If plant specimens were collected from the field, describe the collection location, date and sampling procedures.

Novel plant genotypes

Describe the methods by which all novel plant genotypes were produced. This includes those generated by transgenic approaches, gene editing, chemical/radiation-based mutagenesis and hybridization. For transgenic lines, describe the transformation method, the number of independent lines analyzed and the generation upon which experiments were performed. For gene-edited lines, describe the editor used, the endogenous sequence targeted for editing, the targeting guide RNA sequence (if applicable) and how the editor was applied.

Authentication

Describe any authentication procedures for each seed stock used or novel genotype generated. Describe any experiments used to assess the effect of a mutation and, where applicable, how potential secondary effects (e.g. second site T-DNA insertions, mosiacism, off-target gene editing) were examined.
